# Comparing the Evidence from Observational Studies and Randomized Controlled Trials for Nonskeletal Health Effects of Vitamin D

**DOI:** 10.3390/nu14183811

**Published:** 2022-09-15

**Authors:** William B. Grant, Barbara J. Boucher, Fatme Al Anouti, Stefan Pilz

**Affiliations:** 1 Sunlight, Nutrition and Health Research Center, San Francisco, CA 94164-1603, USA; 2 The London School of Medicine and Dentistry, The Blizard Institute, Barts, Queen Mary University of London, London E1 2AT, UK; 3 Department of Health Sciences, College of Natural and Health Sciences, Zayed University, Abu Dhabi 144534, United Arab Emirates; 4 Division of Endocrinology and Diabetology, Department of Internal Medicine, Medical University of Graz, 8036 Graz, Austria

**Keywords:** breast cancer, colorectal cancer, gestational diabetes, preeclampsia, preterm birth

## Abstract

Although observational studies of health outcomes generally suggest beneficial effects with, or following, higher serum 25-hydroxyvitamin D [25(OH)D] concentrations, randomized controlled trials (RCTs) have generally not supported those findings. Here we review results from observational studies and RCTs regarding how vitamin D status affects several nonskeletal health outcomes, including Alzheimer’s disease and dementia, autoimmune diseases, cancers, cardiovascular disease, COVID-19, major depressive disorder, type 2 diabetes, arterial hypertension, all-cause mortality, respiratory tract infections, and pregnancy outcomes. We also consider relevant findings from ecological, Mendelian randomization, and mechanistic studies. Although clear discrepancies exist between findings of observational studies and RCTs on vitamin D and human health benefits these findings should be interpreted cautiously. Bias and confounding are seen in observational studies and vitamin D RCTs have several limitations, largely due to being designed like RCTs of therapeutic drugs, thereby neglecting vitamin D’s being a nutrient with a unique metabolism that requires specific consideration in trial design. Thus, RCTs of vitamin D can fail for several reasons: few participants’ having low baseline 25(OH)D concentrations, relatively small vitamin D doses, participants’ having other sources of vitamin D, and results being analyzed without consideration of achieved 25(OH)D concentrations. Vitamin D status and its relevance for health outcomes can usefully be examined using Hill’s criteria for causality in a biological system from results of observational and other types of studies before further RCTs are considered and those findings would be useful in developing medical and public health policy, as they were for nonsmoking policies. A promising approach for future RCT design is adjustable vitamin D supplementation based on interval serum 25(OH)D concentrations to achieve target 25(OH)D levels suggested by findings from observational studies.

## 1. Introduction

The year 2022 marks a century since the discovery of vitamin D [1]. The National Library of Medicine’s PubMed database currently includes 97,024 vitamin D–related publications, with 73,577 having “vitamin D” in the title or abstract [accessed August 5, 2022]. Most vitamin D research before 2000 was related to skeletal effects. Interest in nonskeletal effects had become the most important topic of vitamin D research by the year 2000 when there were 69,090 publications related to vitamin D, 59,104 having “vitamin D” in the title or abstract. Despite that vast body of literature, considerable confusion remains regarding vitamin D’s role in determining health outcomes; this mainly results from disagreements between findings from observational studies of health outcome associations with serum 25-hydroxyvitamin D [25(OH)D] concentrations and those from randomized controlled trials (RCTs) of vitamin D supplementation. Findings in 2014 from the two approaches revealed widespread disagreement. Observational studies generally reported inverse correlations between serum 25(OH)D concentrations and health outcomes, whereas RCTs generally showed no significant health benefits of vitamin D supplementation [2]. Meta-analyses of prospective cohort studies of nonskeletal disorders had reported significantly reduced risk for highest versus lowest 25(OH)D concentrations for cardiovascular disease (CVD) incidence and mortality rates, diabetes incidence, colorectal cancer incidence, but not for several other cancers [2]. Because of these discrepancies, Autier and colleagues suggested that observational study findings could be the result of reverse bias—the lowering of 25(OH)D concentrations by illness—and of various biases and confounders inherent to observational study designs [2]. Some conditions do indeed reduce the level of 25(OH)D in serum. However, that conclusion ignores the fact that such reductions limit the supply of 25(OH)D to the tissues, thereby aggravating the effects of vitamin D inadequacy and might also reflect locally increased intracellular calcitriol formation following compensatory increases in intracellular 25(OH)D activating enzyme activity in some tissues [3].

By 2017, RCTs had failed to demonstrate significant nonskeletal benefits for vitamin D except for reductions in common upper respiratory tract infections (RTIs) and in asthma exacerbations [4,5,6]. By April 2019, however, RCTs had shown that vitamin D supplementation had beneficial effects for primary prevention of acute RTIs and reduced acute exacerbations of asthma and chronic obstructive pulmonary disease [7]. The situation deteriorated further in 2019, when results of two large vitamin D RCTs were reported, the VITamin D and OmegA-3 TriaL (*VITAL*) for cancer and CVD risks [8] and the Vitamin D and Type 2 *Diabetes* (*D2d*) trial [9]. Neither trial showed reductions of the disease of interest based on ‘intention to treat’ when comparing disease incidence between the treatment and placebo arms. Neither article’s abstract mentioned the beneficial effects that secondary analyses later reported. Thus, the press reported that vitamin D did not prevent cancer, CVD, or diabetes and medical and public health bodies, media, and the public thought vitamin D’s beneficial effects for nonskeletal health outcomes had been disproven.

Observational studies related to vitamin D are generally prospective cohort or nested case–control studies of health outcomes in relation to baseline serum 25(OH)D concentrations. Participants are enrolled in cohorts or studies and provide blood samples, other biological data, and information on lifestyle upon recruitment. Serum 25(OH)D concentrations are measured later in standardized assays using deep-frozen serum or plasma samples. Generally, no further information (beyond details on health status) is gathered from participants during the follow-up period, which can last from weeks or months to twenty years. At the end of follow-up, changes in health status are correlated with baseline serum 25(OH)D concentrations, with adjustments for data on other relevant factors at enrollment. In nested case–control studies, control subjects may or may not be carefully matched with cases. Case–control studies also can be done with 25(OH)D concentrations measured near the time of health events. However, such studies are unreliable for pre-disease serum 25(OH)D concentrations since disease can affect them; for example, severe infections can reduce 25(OH)D concentrations, an effect that is strongest for acute inflammatory diseases such as acute RTI [10] and is a concern with COVID-19 [10].

Several observational studies now exist based on vitamin D supplementation with measurements of 25(OH)D concentrations at baseline and at follow-up. Conditions studied include breast cancer [11], arterial hypertension [12], myocardial infarction (MI), all-cause mortality rate [13], and preterm birth [14]. According to a recent review [15], such observational studies require careful interpretation in light of their inherent limitations, as discussed later in this review.

Meta-analyses of prospective observational studies of cancer incidence showed significant inverse correlations between baseline serum 25(OH)D concentrations and incidence of 10 different types of cancers between 2016 and 2021 (including bladder, breast, colorectal, head and neck, liver, lung, ovarian, pancreatic, renal, and thyroid cancer) and a direct correlation for prostate cancer (Table 5 in [16]). However, without support from RCTs, such results from observational studies are generally overlooked or ignored.

Data from observational studies of vitamin D obtained from diet have been limited. The most important was that by Garland and colleagues, associating dietary vitamin D and calcium with reduced risk of colorectal cancer [17]. The main problem with using dietary supply values is that dietary sources generally account for only 10–20% of total vitamin D supply. Solar UVB exposure contributes most, as seen from the seasonal variation of serum 25(OH)D concentrations [18]. Meat is also an important source of vitamin D, due to its 25(OH)D content, [19] but 25(OH)D has only recently been included in food frequency tables.

Classically, RCTs are used to evaluate drugs for treatment of disease. People who might benefit from taking the drug of interest are invited to participate and, when enrolled, are randomly assigned to either the treatment or placebo arm. A basic assumption is that no participants obtain the drug outside the RCT. Drug efficacy and adverse effects are the investigated outcomes. Pharmacokinetics of new drugs are also characterized to identify optimum circulating levels of the drug for efficacy and for safety. Safety has often been studied for vitamin D, but the 25(OH) D concentrations needed for efficacy in different disorders have been largely ignored for vitamin D RCT designs until recently [20,21]. Moreover, even in the placebo group in vitamin D RCTs, there are no participants without any vitamin D intake, so that such trials can only compare groups with higher versus lower vitamin D supply.

Uncontrolled intervention studies of vitamin D to prevent dental caries were conducted on adolescents from 1924 to 1945 [22]. Vitamin D RCTs were first conducted in about 1973 for treatment of epileptic patients taking anticonvulsant drugs [23] and became more popular in the early 21st century for studies of disease prevention, being designed in accordance with the guidelines for trials of therapeutic agents, as discussed. 

Most vitamin D RCTs for nonskeletal disorders report no benefits using intention-to-treat analyses [4]. However, problems with such RCTs have included that few participants had any degree of vitamin D deficiency [25(OH)D concentrations < 20 ng/mL] and that moderate, or even unspecified, vitamin D supplementation was permitted in the control or in both study arms. In addition, vitamin D doses were not optimized to achieve any specific target 25(OH)D concentration even though specific thresholds have been identified for many health benefits [15]. Thus, few nonskeletal benefits have been revealed to date from ‘intention to treat’ analyses. However, significant benefits identified have included reduced cancer mortality rates [24], acute RTI risks [25] and autoimmune disease risks [26], as mentioned, along with reductions in several adverse pregnancy outcomes, discussed later.

Heaney outlined guidelines for optimizing design and analysis of clinical studies of nutrients in 2014 [27]:

1. Basal nutrient status must be measured, used as an inclusion criterion for entry into study, and recorded in the report of the trial.
2. The intervention (change in nutrient exposure or intake) must be large enough to change nutrient status and must be quantified.
3. The change in nutrient status produced in trial participants must be measured by validated laboratory analyses and recorded in the report of the trial.
4. The hypothesis to be tested must be that a change in nutrient status (not just a change in diet) produces the sought effect.
5. Conutrient status must be optimized to ensure that the test nutrient is the only nutrition-related, limiting factor in the response.

A version of those guidelines specifically for vitamin D also has been published [28]. No large vitamin D RCTs reported to date for prevention of chronic and infectious diseases have followed these guidelines, partly because most of the trials were designed before 2014. Most RCTs still fail to use 25(OH)D concentrations as a criterion for participation. If they did, enrolling the desired number of participants would be hard. Many RCTs do not measure baseline or achieved 25(OH)D concentrations of any, let alone all, participants. Vitamin D_3_ doses generally range from <1000 to 2000 IU/d and up to ~100,000 IU/month, with some trials using 4000 IU/d. No large vitamin D RCTs have yet optimized co-nutrient status. Magnesium concentration, for example, plays an important role in vitamin D metabolism and affects serum 25(OH)D concentrations upon vitamin D supplementation [29], and magnesium deficiency is common [30]. By contrast, some vitamin D RCTs gave the treatment arm calcium supplements which do not affect 25(OH)D concentrations but can affect health outcomes [31]. For example, calcium supplements but not high dietary calcium intakes may increase the risk of CVD [32].

Pilz and colleagues have also discussed secondary outcomes and subgroup analyses, noting that those analyses reported some beneficial effects of vitamin D supplementation but that they should be considered “explorative outcome” analyses [15]. The case could be made that even though the researchers did not propose looking for such outcomes in the trial design, if good mechanistic data exist to suggest that such results could be expected, they should be considered useful trial findings, especially since, given the large cost and effort needed to conduct large-scale vitamin D RCTs, it is doubtful that any further such trials will be carried out, as discussed later. 

Mendelian randomization (MR) studies examine how genetic variation—typically single-nucleotide polymorphisms (SNPs)—affects health outcomes through the vitamin D pathways. Polymorphisms affecting serum 25(OH)D are used as proxies for long-term vitamin D provision [33]. SNPs are not thought to change in response to behavior or disease experiences, so that they should not be affected by confounding factors and are deemed suitable for assessing causality. However, whether epigenetic effects can modulate the effects of genetic variants throughout adulthood is the subject of ongoing research [34,35,36]. A total of 143 genetic variants associated with vitamin D have been identified in the UK Biobank dataset [37], and recent MR studies have used up to 77 SNPs [38]. Another recent study used 35 SNPs, accounting for 2.8% of the variation in 25(OH)D in the UK Biobank dataset [39]. MR analyses generally require many thousands, often ~100,000 participants, to obtain sufficient statistical power. Whereas linear MR analyses have indicated some significant effects of vitamin D, nonlinear analyses [for different levels of participant vitamin D status] may be more appropriate, since vitamin D effects are non-linear, particularly when J- or U-shaped curves are expected a priori [39,40].

Geographical and temporal ecological studies have been used to identify diseases affected by solar UVB exposure, an index for vitamin D production. Annual solar radiation was shown to be inversely correlated with U.S. colon cancer mortality rates by the brothers Garland in 1980 [41], who hypothesized vitamin D production as the mechanism. Many more ecological studies of cancer incidence and/or mortality rates with respect to indices of solar UVB doses have since been reported [16]. 

Ecological studies are often considered hypothesis generating and, indeed, have led to many further studies exploring vitamin D’s role in reducing cancer risks [16]. Many other diseases also have incidence and/or mortality rates inversely correlated with solar UVB doses: anaphylaxis/food allergy, atopic dermatitis and eczema, attention deficit–hyperactivity disorder, autism, back pain, cancer, dental caries, type 1 diabetes, hypertension, inflammatory bowel disease, lupus, mononucleosis, multiple sclerosis (MS), Parkinson disease, pneumonia, rheumatoid arthritis, and sepsis. [42]; all those diseases have also been linked to low 25(OH)D concentrations. Unfortunately, geographical ecological studies are considered weak, not just being hypothesis generating, but because unmodeled confounders (residual confounding) might explain the findings. However, two ecological studies have looked at cancer mortality rates for whites in the United States with respect to summertime solar UVB doses. The findings with UVB alone [43] were later found to be virtually unchanged after including other cancer risk–modifying factors such as alcohol consumption, Hispanic heritage, lung cancer (as a proxy for smoking and diet), poverty, and degree of urbanization [44]. For diseases where solar UVB doses have been inversely correlated with risk, no non–vitamin D effects are apparent from UVB exposure. However, for diseases with pronounced seasonal variations, such as CVD, hypertension, and RTIs, it appears that UVA-induced increases in serum nitric oxide (NO) as well as changes in temperature could also affect risk [45], though those diseases show no significant inverse correlations with annual UVB doses.

Reconciling differences between observational studies and RCTs regarding vitamin D is important for several reasons. One is that observational studies are generally not considered able to establish causality because some degrees of bias and confounding can never be totally excluded. For example, ‘associations’ could be due to unmodeled factors such as non–vitamin D health benefits of UV exposure [45]. RCTs are generally considered able to show causality and are relied on in medicine and public health policies for guidance but major vitamin D RCTs for nonskeletal effects have not shown any significant effects in primary outcome analyses. By contrast, MR studies are now reporting significant inverse correlations between genetically determined 25(OH)D concentrations and the risk of diseases such as CVD [40]. If observational studies can be shown to determine 25(OH)D concentration–health outcome relationships reliably, at least for some important diseases or outcomes, those studies could provide a basis for public health policies instead of waiting for “proof” of causality from RCTs that may never be carried out, as was the case for lung cancer due to smoking [46].

This review aims to present findings from observational studies of 25(OH)D concentrations and from RCTs of vitamin D supplementation for major health outcomes and to evaluate each approach’s strengths and weaknesses together with brief discussions of results from other approaches, such as MR studies, the mechanisms of vitamin D known to be relevant to each outcome, and ecological studies relevant to possible health effects of vitamin D. The primary health outcomes considered are Alzheimer’s disease (AD) and dementia; autoimmune diseases; cancers; CVD; COVID-19; major depressive disorder (MDD), type 2 diabetes mellitus (T2DM); hypertension; mortality (all-cause); and RTIs, as well as pregnancy and birth outcomes. This review is a narrative rather than a systematic review. It aims to summarize the existing literature regarding some common health outcomes and focuses on comparing findings from different study designs and discussing their strengths and limitations. 

## 2. Results

### 2.1. Diseases and Outcomes

#### 2.1.1. Autoimmune Diseases

In autoimmune diseases, an aberrant immune response is directed against normal human proteins [47]. Calcitriol modulates the immune system through effects on B cells, CD4^+^ and CD8^+^ cells, dendritic cells, innate lymphoid cells, macrophages, and unconventional T cells [48].

By March 2019, observational studies reported an inverse association between vitamin D status and developing autoimmune diseases, such as systematic lupus erythematosus, thyrotoxicosis, type 1 diabetes, MS, iridocyclitis, Crohn’s disease, ulcerative colitis, psoriasis vulgaris, seropositive rheumatoid arthritis, and polymyalgia rheumatica [47].

In the VITAL trial, vitamin D supplementation reduced the risk of incident autoimmune diseases [26]. In the vitamin D arm, 123 participants in the treatment group and 155 in the placebo group developed a confirmed autoimmune disease (hazard ratio [HR] = 0.78 [95% confidence interval (CI), 0.61–0.99; *p* = 0.05]). The incident autoimmune diseases that differ in the raw numbers in the vitamin D versus placebo group were unspecified autoimmune disease (40 vs. 56), polymyalgia rheumatica (31 vs. 43), autoimmune thyroid disease (21 vs. 11), rheumatoid arthritis (15 vs. 24), and psoriasis (15 vs. 23). In this context, viral or bacterial infections, through increasing inflammation, are risk factors for autoimmune thyroid disease [49], rheumatoid arthritis [50], and psoriasis [51]. The finding that vitamin D supplementation reduces autoimmune disease risks can be supported logically, by the ability of vitamin D to reduce the risk of many infections through inducing the secretion of cathelicidin (LL-37) [52] and by reducing inflammation per se [53].

#### 2.1.2. Cancers

Cancers generally arise from genetic mutations of cells leading to tumor formation. The immune system maintains active cell surveillance to evaluate whether they belong in the organs or tissues where they are located. Vitamin D plays an important role in that process, regulating cellular differentiation, progression and apoptosis. Vitamin D also reduces angiogenesis around tumors and reduces the development of metastasis [16,54]. 

The first observational report of serum 25(OH)D and cancer outcome was a 1989 U.S. study [55] over 8 years in Maryland, involving 34 colon cancer cases diagnosed between August 1975 and January 1983 and 67 matched controls from a pool of 25,620 volunteers. The risk of colon cancer decreased with 25(OH)D concentrations above 20 ng/mL: for ranges of 4–19, 20–26, 27–32, 33–41, and 42–91 ng/mL, odds ratios (ORs) were 1.00, 0.48, 0.25, 0.21, and 0.73, respectively. The higher OR for 42–91 ng/mL was probably due to participants’ taking vitamin D supplements, perhaps on medical advice to address concerns about osteoporosis [56]. A recent meta-analysis of colorectal cancer incidence with respect to serum 25(OH)D concentration in prospective studies found an insignificant increase in OR for 25(OH)D above 100 ng/mL compared to 87.5–100 ng/mL [57].

Many similar observational studies have been conducted, generally for colorectal cancer. A 2019 meta-analysis included 12 studies for men and 13 for women. Muñoz and Grant [16] point out that the authors of that meta-analysis did not consider or adjust the results for follow-up time. Thus, the reduced risk for higher 25(OH)D concentrations would have been underestimated, leading to the conclusion that men who developed colorectal cancer derived no benefit [57]. As shown in Figure 1 of [16], a linear regression fit to OR versus follow-up time had the equation OR = 0.74 + 0.031*x* years (*r* = 0.79) for men and OR = 0.77 + 0.081*x* years (*r* = 0.25) for women. Thus, adjusting the reported values for study duration could have yielded similar beneficial effects for men as for women because the apparent reductions decline with increasing follow-up time, probably due to changes in serum 25(OH)D concentrations [58,59,60]. The 2015 analysis showed that the regression fitted to the prospective observational studies of breast cancer incidence over time had an association for time zero, by extrapolation, that corresponded well with results from case–control studies in which 25(OH)D had been measured near the time of diagnosis [60].

An observational study of breast cancer incidence based on analysis of individual participant data from 3325 participants in two vitamin D RCTs [31,61] and 1713 participants in the GrassrootsHealth (GRH) prospective cohort study was reported in 2018 [11]. Serum 25(OH)D concentrations were measured at baseline and after a year of follow-up in the RCTs and every 6 months in the GRH cohort. Participants in the treatment arms of the RCTs took 1100 IU/d of vitamin D_3_ plus 1450 mg/d of calcium in the first Lappe study and 2000 IU/d of vitamin D_3_ plus 1500 mg/d of calcium in the second Lappe study while participants in the GRH study chose their own vitamin D supplemental intake. A total of 77 women developed breast cancer during the study periods, of whom 14 were from the GRH cohort. Women who achieved a 25(OH)D concentration >60 ng/mL compared with those achieving <20 ng/mL had a large risk reduction: HR = 0.20 (95% CI, 0.05–0.82, *p* = 0.03; *p*_trend_ = 0.04) after adjustment for age, body mass index (BMI), smoking status, and calcium supplement intake. That study’s strengths included that many participants were from vitamin D RCTs, that all had 25(OH)D concentrations measured at baseline and follow-up, and that 25(OH)D concentrations ranged from 10 to 75 ng/mL.

##### Cancers—RCTs

The *VITAL* study was an ambitious project aiming to determine whether vitamin D and omega-3 supplementation reduced cancer rates, CVD incidence, and mortality rates [8]. VITAL enrolled 25,871 participants, including 18,046 white people, 5106 African Americans, and 2719 people of other or unknown race or ethnic group. Mean (SD) age was 67 ± 7 years, mean (SD) BMI was 28 ± 6 kg/m^2^, and mean (SD) baseline 25(OH)D concentrations in the vitamin D treatment group were 28 ng/mL for 395 males and 32 ng/mL for 441 females as determined from using blood samples collected mainly in winter or spring. Participants were recruited between November 2011 and March 2014, and the intervention ended December 31, 2017, yielding a median follow-up period of 5.3 years. Participants in the vitamin D treatment arm took 2000 IU/d of vitamin D_3_. However, all participants up to 70 years of age were permitted to take additional amounts of up to 600 IU/d of vitamin D and up to 800 IU/d if aged over 70 years.

The VITAL study did not show that supplementing with 2000 IU/d of vitamin D_3_ reduced risk of incident cancer according to intention-to-treat analyses [8]. However, in secondary analyses, people with BMI < 25 kg/m^2^ had a reduced risk (HR = 0.76 [95% CI, 0.63–0.90]). The baseline and achieved 25(OH)D concentrations for those participants with such data gave values of 33.3 and 45.9 ng/mL, respectively. The difference between baseline and achieved 25(OH)D concentration was 12 ng/mL for three BMI categories (healthy weight, <25; overweight, 25 to <30; and obese, ≥30 kg/m^2^). Evidently, the vitamin D supplementation used did not counter obesity-related inflammation, because obesity increases vitamin D requirements [62]. In a 26-week RCT involving 52 participants aged 18–50 years given 7000 IU/d of vitamin D_3_, mean serum 25(OH)D concentrations increased from 13 to 44 ng/mL, but inflammatory markers including high-sensitivity C-reactive protein (hsCRP) were unaffected [63]. Chronic inflammation is an important cancer risk factor and meta-analyses haves associated CRP with breast cancer (HR = 1.14 [95% CI, 1.01–1.28]; OR = 1.23 [95% CI, 1.05–1.43]); colorectal cancer (OR = 1.34 [95% CI, 1.11–1.59]); and lung cancer (HR = 2.03 [95% CI, 1.59–2.50]) [64].

Why have RCTs not shown that vitamin D supplementation reduces risk of cancer incidence? That could be explained in three ways:

1. With findings based on vitamin D dose rather than achieved 25(OH)D concentration, enrolled participants would include those with relatively high 25(OH)D concentrations, lowering the chances of detecting reduced cancer incidence.
2. Higher 25(OH)D concentration lower cancer mortality risks more strongly than it reduces cancer incidence rates [24].
3. Vitamin D simply has no significant effect on cancer incidence.

Table 1 shows that vitamin D’s effect was always stronger for cancer mortality rate than incidence rate in studies comparing those outcomes.

One narrative review includes 25 papers (8 RCTs on cancer patients, 8 population RCTs, and 9 observational studies) found through March 2021, published between 2003 and 2020. That review revealed some evidence that vitamin D supplementation in cancer patients could improve cancer survival, but no significant effect was reported in RCTs [68]. Some observational studies reported evidence associating vitamin D supplementation with increased survival among cancer patients, and only one study indicated an opposite effect. Those findings, therefore, were inconclusive. 

Prospective or retrospective cohort studies evaluating the association between blood 25(OH)D level and survival outcomes in women with breast cancer were included in another review [69]. Outcome measures included overall survival, breast cancer–specific survival, and disease-free survival. Twelve studies involving 8574 female breast cancer patients were identified and analyzed. In comparing the lowest with highest category of baseline 25(OH)D level, the pooled adjusted HR was 1.57 (95% CI, 1.35–1.83) for overall survival, 1.98 (95% CI, 1.55–2.53) for disease-free survival, and 1.44 (95% CI, 1.14–1.81) for breast cancer–specific survival.

##### Cancers—Geographical Ecological Studies

Geographical ecological studies strongly support the UVB–vitamin D–cancer hypothesis as proposed in 1980 by the brothers Garland after comparing the U.S. colon cancer mortality rate map with the map of annual solar radiation [41]. In the latest review, four single-country or region ecological studies published between 2006 and 2012 reported inverse correlations between indices of solar UVB doses for incidence rates for 21 cancers. Furthermore, five single-country ecological studies published between 2006 and 2011 reported inverse correlations between indices of solar UVB doses and mortality rates for 24 different types of cancer [16].

The Centers for Disease Control and Prevention continues to post maps of cancer incidence rates averaged by state (https://gis.cdc.gov/Cancer/USCS/#/AtAGlance/, accessed 21 June 2022). The most recent maps are for 2019 and are similar to those in the *Atlas of Cancer Mortality Rates in the United States*, *1950–94* [70]. A comparison of the lung cancer incidence maps with maps of U.S. obesity prevalence shows a high correlation between states with the highest obesity rates and states with the highest lung cancer rates. That finding is consistent with the finding that inflammation is an important risk factor for lung cancer [64] and that vitamin D does not reduce the raised CRP associated with obesity [63]. Similar findings are reported for colorectal cancer but not for female breast cancer [64]. Recent MR studies using nonlinear methodology do show significant effects of higher genetically determined vitamin D status in reducing CRP in deficient subjects [40].

Geographical ecological studies are based on indices of solar UVB doses. A concern is that solar UVB could be an index for more than one mechanism of solar UV, though that is considered unlikely. In support, a recent review examined the role of three UV mechanisms related to the seasonality of CVD, hypertension, and infectious diseases [45]. Reasonable evidence exists that solar UVB has non–vitamin D effects and that solar UVA increases serum NO concentrations, both of which can contribute to the seasonality of several diseases. However, cancers have little seasonal variation [71], and the mechanisms by which vitamin D reduces cancer risk are well known [16]. Thus, geographical ecological studies should be considered a strong support for the UVB–vitamin D–cancer hypothesis.

Such ecological studies are useful for several reasons:

- They are easy to conduct because they can be based on publicly available data.
- They include many participants.
- No participants are omitted.
- The analysis can include many other cancer risk–modifying factors averaged at the population level.
- They can be used to locate cancer hot spots globally.
- Analyses can be performed for different ethnicities and races and can be repeated for different periods.

##### Cancer—Mendelian Randomization Study

A 2021 MR study of genetically predicted cancer incidence used up to 77 independent SNPs for 25(OH)D, representing about 4% of the normal phenotypic variation in serum 25(OH)D, based on analysis of more than 400,000 UK Biobank participants [38]. Various cancer datasets with a genome-wide association study dataset were used. The total number of cancer cases included 122,977 for breast cancer, 25,509 for epithelial ovarian cancer, 12,906 for endometrial cancer, 79,148 for prostate cancer, 12,874 for melanoma, and 10,279 for esophageal cancer. The only cancer with a statistically significant result for increased genetically determined 25(OH)D was epithelial ovarian cancer, with an adjusted odds ratio (aOR) of 0.89 (95% CI, 0.82–0.96). Findings for endometrial, lung, mucinous, neuroblastoma, and pancreatic cancers showed aORs < 1.00 but were not statistically significant. The aORs for skin cancers and melanoma were in the expected direction: melanoma, 1.05 (95% CI, 0.90–1.23); squamous cell carcinoma of the skin, 1.02 (95% CI, 0.88–1.19); and basal cell carcinoma of the skin, 1.16 (95% CI, 1.04–1.28). The aOR for prostate cancer also was in the same direction, 1.11 (95% CI, 0.93 to 1.33). However, that MR study probably failed to detect significant associations between genetically determined 25(OH)D concentrations and cancer risk, having too few cancer cases, and since the genetic variant size effect on serum 25(OH)D values was no larger than the variance of the 25(OH)D assays used at time of measurement.

#### 2.1.3. Cardiovascular Disease

Vitamin D could reduce CVD risk by reducing risks of vascular inflammation, endothelial dysfunction, proliferation of smooth muscle cells, hypertension, and secondary hyperparathyroidism [72]. Vitamin D also has several mechanisms to lower risk of metabolic syndrome [72], T2DM [73], seasonal influenza [74], and periodontal disease [75], that are all risk factors for CVD.

In the Framingham Offspring Study [76], serum 25(OH)D concentrations were measured in 1739 eligible participants between 1996 and 2001 followed up for a mean of 5.4 years. For the 688 hypertensive participants who developed a first CVD event, the fully adjusted HRs, compared with participants with 25(OH)D ≥ 15 ng/mL, were 2.07 (95% CI, 1.19–3.67) for 25(OH)D from 10 to <15 ng/mL and 2.43 (95% CI, 1.23–4.80) for those with 25(OH)D < 10 ng/mL; *p*_trend_ = 0.003. For those without hypertension, no significant differences were found. 

A 2017 meta-analysis looked at total CVD events with respect to baseline 25(OH)D concentrations among 180,667 participants across 34 publications between 2008 and 2015 [77]. The RRs for an increase of 25(OH)D of 10 ng/mL varied as a function of follow-up time: for <5 years, RR = 0.84 (95% CI, 0.78–0.90); for 5 to <10 years, RR = 0.88 (95% CI, 0.77–1.01); and for ≥10 years, RR = 0.92 (95% CI, 0.89–0.96). The linear fit to the RR is RR = 0.81 + 0.0084*x* years. Again, the longer the follow-up, the lower the apparent beneficial effect of higher baseline 25(OH)D concentration. Figure 2 in that review showed that the RR varied from 1.00 at 0 ng/mL to 0.8 ± 0.02 at ~20 ng/mL. 

**Figure 1:**
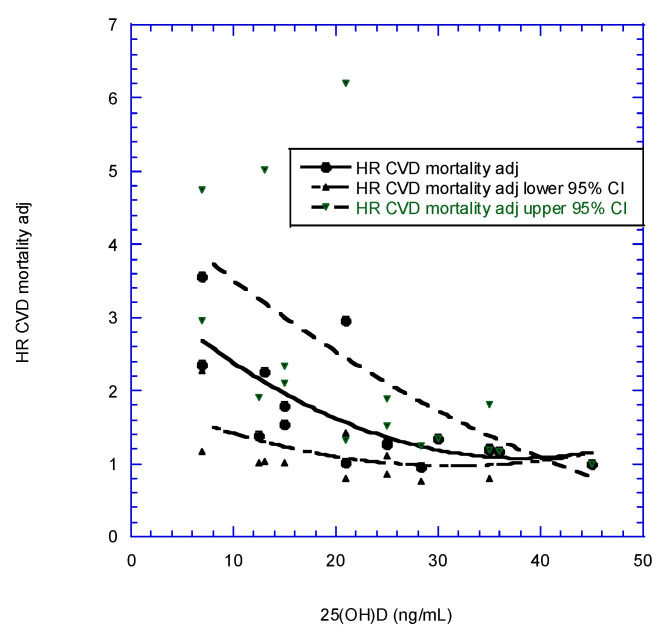
Hazard ratio (HR) and 95% confidence interval (CI) for CVD mortality according to mean serum 25-hydroxyvitamin D [25(OH)D] concentrations for data from four prospective observational studies.

Data from four prospective studies of CVD mortality rate versus baseline serum 25(OH)D concentrations were used to generate a graphical meta-analysis of HR versus 25(OH)D concentration for CVD mortality rate (Table 2 and Figure 1). The HR values were adjusted so that the aHR adjusted value at the highest 25(OH)D concentration for each study fell on the second-order fit to the data [78]. Thus, vitamin D is apparently more effective at reducing risks of CVD mortality than at reducing incidence rates, in general agreement with the finding for all-cancer incidence and mortality rates.

A recent analysis of data from the UK Biobank dataset reported a linear inverse relationship between adjusted 25(OH)D concentration and odds of an incident CVD event [40]. The odds were 1.08 ± 0.03 at 4 ng/mL, 1.0 at 20 ng/mL, and 0.88 ± 0.03 at 52 ng/mL of 25(OH)D. Figure 3 in that article suggests that correcting 25(OH)D to above 20 and 40 ng/mL could reduce CVD incidence rates in the UK by 4% ± 2% and 6% ± 4%, respectively.

A long-term follow-up study of patients treated at the U.S. Veterans Health Administration from 1999 to 2018 showed reduced risk of MI for people supplementing with vitamin D [13]. The patients followed up were those with 25(OH)D concentration < 20 ng/mL at baseline. Many were counseled to take vitamin D supplements. For patients who achieved >30 ng/mL, the HR for MI was 0.65 (95% CI, 0.49–0.85) compared with those who achieved 20–30 ng/mL and 0.73 (95% CI, 0.55–0.96) as compared with those with 25(OH)D < 20 ng/mL. That effect is higher than that found in the UK Biobank study but only applied to MI. That study used propensity scores to correct for potential systematic differences between comparison groups. Included covariates were age, sex, BMI, hypertension, diabetes, coronary artery disease, congestive heart failure, peripheral arterial disease, chronic kidney disease, chronic obstructive pulmonary disease, smoking, concomitant therapies (aspirin, statin, and beta-blockers), and low-density lipoprotein cholesterol levels. The researchers also used propensity score–weighted, stabilized inverse probability of treatment weights to obtain unbiased estimates of treatment effects; hence strengthening the design and analysis. The propensity score is the probability of treatment assignment conditional on observed baseline characteristics and allows designing and analyzing an observational (nonrandomized) study so that it mimics some particular characteristics of an RCT [82].

**Figure 2:**
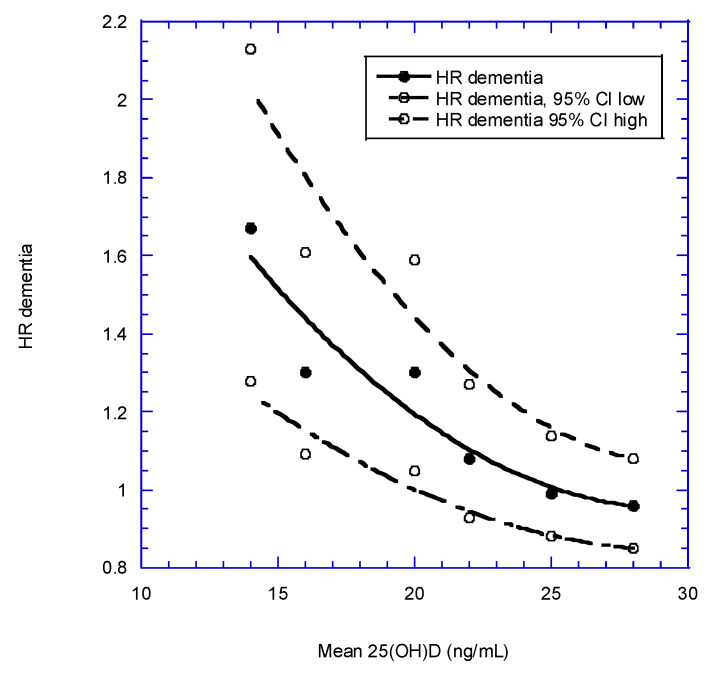
Hazard ratio (HR; mean and 95% confidence interval [CI]) for dementia according to mean 25-hydroxyvitamin D [25(OH)D] concentration in each of the six studies included in the meta-analysis [138].

Parathyroid hormone (PTH) plays an important role in CVD risk. Several observational studies report independent increases in risk for CVD for low 25(OH)D concentrations and high PTH concentrations [83,84,85]. Elevated PTH but not vitamin D deficiency has been associated with increased risk of heart failure [86]. An explanation for the difference in the relative contributions of 25(OH)D deficiency and higher PTH concentrations is that the PTH–25(OH)D relationship changes over time, as this ratio changes with age [87]. Figure 2 in that article shows median PTH values as a function of 25(OH)D concentration for ages < 20, 20–40, 40–60, and >60 years. When the PTH value of 65 pg/mL is used as the threshold concentration for significantly increased CVD events [84], the value is exceeded, with 25(OH)D concentrations of 7 ng/mL for those aged 40–60 years and of ~14 ng/mL for those > 60 years. This PTH effect may also help explain why CVD rates increase with age as well as why they are higher in winter than in summer [45]. 

A meta-analysis of observational studies of risk of atrial fibrillation with respect to serum 25(OH)D concentration as a continuous variable reported higher 25(OH)D concentrations associated with lower risk: OR = 0.96 (95% CI, 0.83–1.00; *p* = 0.04) from five cohort studies and 0.85 (95% CI, 0.79–0.92; *p* <0.0001) from four case–control studies [88]. 

##### Cardiovascular Disease—RCTs

The VITAL study did not find that vitamin D supplementation reduced overall CVD risks [8], most likely because few participants had baseline 25(OH)D concentrations below 20 ng/mL (only 68/502 in the vitamin D treatment and placebo arms combined). A recent MR study however, clearly indicated that CVD risk decreases rapidly with increasing 25(OH)D concentration in those with baseline vitamin D inadequacy, [40] though the outcomes were not analyzed with respect to achieved 25(OH)D, PTH or season of the CVD event. However, half the participants had hypertension treated with medication and treating hypertension can significantly reduce CVD burden [89] and overall mortality rate [90].

##### Cardiovascular Disease—Mendelian Randomization

The MR study just mentioned showed that genetically predicted 25(OH)D concentrations correlated inversely with CVD risk [40], based on 44,510 CVD cases and 251,269 controls from the UK Biobank; it succeeded because the researchers calculated genetically instrumented 25(OH)D concentrations (as estimated using the 40 principal genetic factors affecting serum 25(OH)D) for each of 100 strata of baseline serum 25(OH)D concentration. Individual participants with 25(OH)D of 10 ng/mL had an OR of 1.11 (95% CI, 1.05–1.18) vs. 20 ng/mL. Individual participants with 25(OH)D of 30 ng/mL had an OR of 0.98 (95% CI, 0.97–0.99) vs. those at 20 ng/mL. The OR for the lowest genetically instrumented 25(OH)D concentration (4 ng/mL) was ~2.3 (95% CI, 1.4–3.5); their reductions in CVD risk reached 6% ± 3% as baseline 25(OH)D concentrations rose to 40 ng/mL.

The mechanisms by which vitamin D might reduce CVD risks are well understood. A recent meta-analysis reported inverse associations of vitamin D status with risks of metabolic syndrome and obesity, BMI, dyslipidemia, blood pressure (BP), insulin resistance, and dysglycemia. Meta-analysis of data from seven RCTs reported that supplementation reduced BP, abdominal obesity, and insulin resistance—all recognized CVD risk markers [63]. Mechanistic evidence shows that vitamin D reduces inflammation, an important factor in the progression of atherosclerosis. Vitamin D also lowers serum triglyceride levels and reduces the secretion of matrix metalloproteinase enzymes 2 and 9, which macrophages release when infiltrating arterial plaque, a process causing the plaque disruption that leads to acute arterial events through overlying clot formation [91]. Such findings are supported by the adverse effects of vitamin D deficiency seen experimentally on the vasculature [92]. 

#### 2.1.4. COVID-19

Grant and colleagues suggested (April 2020) that higher 25(OH)D concentrations should reduce risk of COVID-19 incidence and death [93]. As of 16 June 2022, more than 2100 publications were found at pubmed.gov by searching for “vitamin D, COVID-19”. Most observational reports were case–control studies with 25(OH)D measured around the time of a SARS-CoV-2–positive PCR test or COVID-19 symptoms. 

Vitamin D’s mechanisms to reduce risk of SARS-CoV-2 infection and COVID-19 incidence, severity, and death are now well known. These include reduced viral replication through inducing cathelicidin (LL-37) and reduced risk of the cytokine storm [93,94]. 

A large database of test results for SARS-CoV-2 positivity for patients who had serum 25(OH)D concentrations measured during the 12 months preceding the positive test by Quest Diagnostics between 9 March and 19 June 2020 was examined [95]. The 25(OH)D concentrations were seasonally adjusted, with a value of 32 ± 12 (mean ± SD) ng/mL. As analyzed by race/ethnicity, for white, non-Hispanic patients, SARS-CoV-2 positivity declined from 9% for 25(OH)D < 20 ng/mL to 5% for 60 ng/mL; for Hispanic patients, from 16% for <20 ng/mL to 8% at 50 ng/mL and from 19% for <20 ng/mL to 10% at 25(OH)D > 55 ng/mL in Black non-Hispanic subjects. However, such studies can be confounded by factors linked to both low circulating 25(OH)D concentrations and more severe COVID-19 [96].

More recently, two observational studies reported COVID-19 risk for people using vitamin D supplementation. One was from Barcelona, using data for vitamin D prescriptions and risk of SARS-CoV-2 or COVID-19 [97]. Most identifiable vitamin D use is by prescription rather than over-the-counter, though current usage is probably mainly over-the-counter. Patients on cholecalciferol treatment achieving 25(OH) D concentrations ≥30 ng/mL had lower risk of SARS-CoV2 infection, lower risk of severe COVID-19, and lower COVID-19 mortality than supplemented 25(OH)D–deficient patients (HR = 0.66 [95% CI, 0.46–0.93]; *p* = 0.02). Patients on calcifediol treatment achieving serum 25(OH)D concentrations ≥ 30 ng/mL had lower risks of SARS-CoV2 infection, of severe COVID-19, and of COVID-19 mortality than 25(OH)D–deficient patients not supplementing with vitamin D (HR = 0.56 [95% CI, 0.42–0.76]; *p* < 0.001).

The second study was based on 4599 veterans in U.S. Department of Veteran Affairs health care facilities. Participants received a positive SARS-CoV-2 test and a blood 25(OH)D test between 20 February 2020, and 8 November 2020, and were monitored for up to 60 days. After adjustment for all covariates, including race/ethnicity and poverty, a significant independent inverse dose–response relationship was evident between increasing 25(OH)D concentrations (from 15 to 60 ng/mL as a continuous variable) and decreasing probability of COVID-19–related hospitalization (from 24.1% to 18.7%; *p* = 0.009) and mortality (from 10.4% to 5.7%; *p* = 0.001) [98].

COVID-19 outcomes with respect to serum 25(OH)D concentrations from meta-analyses based on 72 observational studies published through 30 May 2021, were reported recently [99]; vitamin D deficiency/insufficiency increased odds of developing COVID-19, severe COVID-19, and death. Mean 25(OH)D concentrations were 4–5 ng/mL lower in people with COVID-19 than in controls for all outcomes. Associations between vitamin D deficiency/insufficiency and death were insignificant when studies with high risk of bias or reporting unadjusted effect estimates were excluded but bias and heterogeneity risks were high across all analyses. Discrepancies in timing of vitamin D testing, definitions of severe COVID-19, and of vitamin D deficiency/insufficiency contributed to that heterogeneity while serum 25(OH)D concentrations fall with severe COVID-19 illness [10], though whether that effect might increase the health benefits of adequate supplementation is a postulate urgently needing to be tested. 

A meta-analysis of vitamin D supplementation on COVID-19–related outcomes from publications through January 2022 reported significantly reduced risk of admission to intensive care units (RR = 0.35 [95% CI, 0.20–0.62]) and reduced mortality (RR = 0.46 [95% CI, 0.30–0.70]) [100], though vitamin D status had no significant independent effect on COVID-19 incidence. Recently, positive effects of vitamin D supplementation on hospitalized COVID-19 patients were reported [101], adjuvant supplementation reducing hospital stay and duration of oxygen requirement.

Given that observational studies on the potential effectiveness of vitamin D supplementation for the prevention and treatment of COVID-9 are justifiably criticized for their limitations, inherent to observational study designs, readers should note that several public health recommendations during the COVID-19 pandemic, such as for the fourth SARS-CoV-2 vaccine dose, were also based solely on observational data and risk to benefit estimates but not on RCTs. Results from vitamin D RCTs are limited and inconsistent for COVID-19, so that no final conclusion can be drawn to date on the value of vitamin D supplementation regarding COVID-19.

#### 2.1.5. Diabetes Mellitus Type 2

T2DM usually develops after a long period of increased insulin resistance (IR) where increased insulin concentrations become necessary to activate insulin effects in tissues such as liver and muscle. The demand for increased insulin secretion leads to islet beta cell damage and eventual inadequacy of insulin secretion with resultant hyperglycemia. Calcitriol is essential for normal insulin secretory responses to glucose and reduces the abnormal hepatic production of glucose and triglycerides seen in IR. Vitamin D effects also suppress inflammatory processes active in IR that contribute to the increased risks of both T2DM, and CVD seen with IR [102,103,104,105]. Vitamin D reduces inflammation and regulates intracellular Ca^2+^ level in many cell types, including islet beta cells and hepatocytes, contributing to reduced IR as reviewed by Szymczak-Pajor and colleagues [106].

Observational studies have long reported that 25(OH)D concentrations are inversely correlated with T2DM and with features of the metabolic syndrome [107]. A 2007 meta-analysis noted that for prevalence of T2DM in non-black people, the OR for highest versus lowest 25(OH)D concentration was 0.71 (95% CI, 0.57–0.89) [108]. A 2013 meta-analysis of 21 prospective studies involving 76,220 participants and 4996 incident T2DM cases showed that for each 4-ng/mL increase in 25(OH)D concentration, risk of T2DM decreased by 4% (95% CI, 3–6%), with the lowest risk near 60 ng/mL [109]. That analysis was updated by adding studies published up to August 31, 2016 [110]. With data from 31 (nested) case–control and cohort studies comparing participants with 25(OH)D values of approximately 20–30 ng/mL with those in the lowest category, the OR was 0.77 (95% CI, 0.72–0.82). With 23 (nested) case–control and cohort studies comparing participants with the highest 25(OH)D concentrations with those with the lowest 25(OH)D category, the OR was 0.66 (95% CI, 0.61–0.73). Again, reduced T2DM risks were seen up to 25(OH)D concentrations of ~50–60 ng/mL.

##### Diabetes Mellitus Type 2—RCTs

The *D2d* trial evaluated whether vitamin D supplementation could reduce risk of progressing from prediabetes to T2DM [9] and enrolled 2423 prediabetic participants, mean (SD) age 60 ± 10 years. Participants mean (SD) 25(OH)D concentration was 28 ± 10 ng/mL. Half were randomized to take 4000 IU/d of vitamin D_3_ and half to take a placebo during a mean time of 2.5 years. On the basis of intention to treat, the HR for vitamin D compared with placebo was 0.88 (95% CI, 0.75–1.04; *p* = 0.12). In secondary analyses of this dataset, participants with BMI < 30 kg/m^2^ had reduced T2DM risk, HR = 0.71 (95% CI, 0.53–0.95) as did those not given calcium supplements, HR = 0.81 (85% CI, 0.66–0.98). 

A further secondary analysis of D2d data was then made, based on intratrial 25(OH)D concentrations [111]. For participants in the vitamin D treatment arm, each 10-ng/mL increase in 25(OH)D concentration above 20–30 ng/mL up to >50 ng/mL was associated with a significant HR of 0.75 (95% CI, 0.68–0.82) for progression to diabetes. No effect was seen in the placebo arm. Those findings strongly support the Heaney guidelines for basing analyses of vitamin D RCTs on achieved 25(OH)D concentrations, and show the importance of assessing vitamin D’s effects on health by vitamin D status, not by dosages [27].

##### Diabetes Mellitus Type 2—Mendelian Randomization

Two MR analyses found that genetic variants of 25(OH)D were causally linked to risk of T2DM. The first one was reported in 2018 [112]. It used data from the China Kadoorie Biobank as well as other studies. A 10-ng/mL higher biochemically measured 25(OH)D was associated with a 9% (95% CI: 0–18%) lower risk of diabetes in the China Kadoorie Biobank. In a meta-analysis of all studies, a 10-ng/mL higher genetically instrumented 25(OH)D concentration was associated with a 14% (95% CI: 3–23%) lower risk of diabetes (*p* = 0.01) using two synthesis SNPs. An equivalent difference in 25(OH)D using a genetic score with 4 SNPs was not significantly associated with diabetes (odds ratio 8%, 95% CI: −1% to 16%, lower risk, *p* = 0.07), but had some evidence of pleiotropy. 

A second MR analysis was performed using data on genetic variants for 25(OH)D from a genome-wide association study on UK Biobank subjects [*n* = 329,247] of European ancestry. A higher genetically instrumented 25(OH)D was causally linked to reduced risk of T2DM (OR per standard deviation increase in 25(OH)D = 0.95 [95% CI, 0.91–0.99]; *p* = 0.01) [113]. That study also confirmed vitamin D’s causal role by studying two SNPs of the vitamin D–activating enzyme, where the HR for each 1-SD increase in 25(OH)D = 0.89 (95% CI, 0.82–0.98; *p* = 0.02). 

#### 2.1.6. Hypertension

Hypertension is generally defined by a systolic BP (SBP) >140 mmHg and a diastolic BP (DBP) > 90 mmHg. One risk causal factor for hypertension is endothelial dysfunction [114] that is related to lower availability of NO, an important vasodilator. Vitamin D can reduce endothelial dysfunction by suppressing renin production, which reduces activity of the renin–angiotensin–aldosterone system and increases expression of endothelial NO synthase (eNOS) [115]. Vitamin D also reduces production of reactive oxygen species and cyclooxygenase 1 (COX-1) mRNA and protein expression [116], thereby reducing production of endothelium-derived vasoconstrictive factors [117]. 

Researchers have found inversely correlated serum 25(OH)D concentrations with BP, starting with cross-sectional studies in 2005 [118] and nested case–control studies in 2007 [119]. Forman and colleagues included 613 men from the Health Professionals Follow-Up study and 1198 women from the Nurses’ Health Study with measured baseline 25(OH)D concentrations who were followed up for 4–8 years. During the 4-year period of follow-up, 61 men and 129 women developed hypertension. The multivariable RRs comparing lowest with highest deciles of 25(OH)D were 2.31 (95% CI, 2.03–2.63) in men and 1.57 (95% CI, 1.44–1.72) in women.

A meta-analysis including 10 prospective studies from 2007 to 2015 reported that a linear increase of each 10 ng/mL in 25(OH)D resulted in an RR of 0.95 (95% CI, 0.90–1.00) [120]. 

Another meta-analysis of dose–response curves for hypertension versus 25(OH)D concentrations from 11 prospective cohort studies showed that the RR at 6 ng/mL versus 29 ng/mL = 1.37 (95% CI, 1.13–1.65). The RR decreased quasi-linearly up to 29 ng/mL before plateauing with 25(OH)Ds of ~50 ng/mL, where the RR was approximately 0.9 (95% CI, 0.8–1.0) [121]. The same meta-analysis found no effect for vitamin D supplementation on BP from the analysis of 27 RCTs.

A Canadian open-label vitamin D supplementation study looked at how raising serum 25(OH)D concentrations affected BP and hypertension [12]; the 8155 participants were given free vitamin D_3_ and other supplements and counseled on how to achieve a 25(OH)D concentration > 40 ng/mL, with medically supervised dose adjustments. Of 592 participants hypertensive at enrollment, 71% were no longer hypertensive 12 ± 3 months later. Their mean (SD) SBP dropped by 18 ± 19 mmHg (95% CI, −24 to −12 mmHg) if not taking BP medications or by 14 ± 21 mmHg (95% CI, −18 to −9 mmHg) if taking BP medications after joining the program. Decreases in DBP were similar. However, for patients not hypertensive at enrollment, decreases in both SBP and DBP were insignificant. Though an observational study, the quality appears to be higher than that of many vitamin D RCTs. For example, many parameters were evaluated at baseline and tabulated by 25(OH)D concentration in 20-ng/mL increments. Values for most parameters, including SBP and DBP, did not vary significantly with baseline 25(OH)D concentration < 60 or 80 ng/mL. At the end of 1 year, mean (SD) serum 25(OH)D concentration had increased from 35 ± 15 ng/mL to 45 ± 16 ng/mL, but mean SBP, DBP, and pulse pressure were unchanged. After correction for possible confounding factors, only participants who were vitamin D insufficient (20–30 ng/mL) at baseline and achieved 25(OH)Ds > 40 ng/mL at follow-up had a lower risk of hypertension (OR = 0.10, 95% CI, 0.01–0.87; *p* = 0.03). 

An unconsidered factor for BP risk is that solar UVA induces release of NO into the circulation, which can significantly lower BP as reported in 2014 by a team led by Weller [122]. The researchers showed that UVA irradiation of the skin lowers BP, that UVA increases nitrite production and reduces circulating nitrate, and that UVA increases blood flow in the forearm, the vasodilator NO being released by photolysis of nitrite to nitrate.

##### Hypertension—RCTs

Several vitamin D RCTs have looked at BP and hypertension. One studied black patients during the winters of 2008 and 2010 [123]. A total of 283 black people with median age 51 years were enrolled with a median 25(OH)D concentration of 16 ng/mL (interquartile range [IQR], 11–23 ng/mL), median SBP of 122 mmHg (IQR, 110–136 mmHg), and median DBP of 78 mmHg (IQR, 71–86 mmHg). Participants were assigned to four groups for 3 months of treatment in ~equal numbers for placebo, 1000, 2000, or 4000 IU/d of vitamin D_3_ daily. The 3-month change in SBP per 1000 IU/d of vitamin D_3_ given was −1.4 ± 0.7 mmHg (mean ± SE); *p* = 0.04, whereas that for DBP was −0.5 ± 0.5 mmHg.

Another vitamin D RCT regarding BP was conducted in Austria between June 2011 and August 2014 [124]. It enrolled 200 hypertensive participants with mean (SD) age 60 ± 11 years and 25(OH)D concentrations below 30 ng/mL. Half of the patients were assigned to the vitamin D treatment arm and received 2800 IU/d of vitamin D_3_ for 8 weeks, whereas the other half received a placebo. The mean (SD) change in SBP was −0.4 (95% CI, −2.8 to 1.9) mmHg (*p* = 0.71). Data in this study [124] were reanalyzed for achieved 25(OH)D concentration [125], suggesting reduced SBP for high versus low 25(OH)D concentrations between 5 and 55 ng/mL (threshold of ~23 ng/mL). However, changes in this very short trial were not statistically significant.

##### Hypertension—Mendelian Randomization

An MR study regarding vitamin D and BP and hypertension was published in 2014 [126]. In a meta-analysis of data from 142,255 participants, the synthesis score for genetic increases in 25(OH)D was associated with a reduced risk of hypertension (OR per allele = 0.98 [95% CI, 0.96–0.99]; *p* = 0.001). In instrumental variable analyses, each 10% increase in genetically instrumented 25(OH)D concentration was associated with a change in SBP of −0.37 mmHg (95% CI, −0.73 to 0.003; *p* = 0.052), in DBP of −0.29 mmHg (95% CI, −0.52 to −0.07; *p* = 0.01), and an 8.1% decrease in hypertension (OR = 0.92 [95% CI, 0.87–0.97]; *p* = 0.002).

#### 2.1.7. Mortality, All-Cause

All-cause mortality rate is mainly due to deaths from CVD, T2DM, respiratory disease, AD and other dementias, autoimmune diseases, and cancer, all of whose mortality rates are inversely correlated with serum 25(OH)D concentrations. Meta-analyses of prospective observational studies also report significant inverse correlations with all-cause mortality rates. One review involved 13 studies published between 2006 and 2010, including 5562 deaths among 62,548 participants [127]. That study showed a significant inverse relationship for 25(OH)D concentrations and mortality below 20 ng/mL. A second meta-analysis based on 32 studies published pre-2013 reported a similar cutoff value of 30 ng/mL [128]. A European individual participant data meta-analysis with 26,916 participants, of whom 6802 died during the median follow-up of 10.5 years, indicated a significant inverse correlation between baseline 25(OH)D concentration and all-cause mortality rates for values < 20 ng/mL [129]. The observational study based on veterans treated by the U.S. Veterans Administration Health System reported a HR of 0.61 (95% CI, 0.56–0.67) for all-cause mortality rate for participants who achieved 25(OH)Ds >30 ng/mL vs. those remaining at <20 ng/mL during 20 years of follow-up [13]. An MR study showed significant inverse correlations between genetically determined 25(OH)D concentrations and all-cause mortality rate [130], as a more recent study also reports [131].

#### 2.1.8. Respiratory Tract Infections

Vitamin D reduces the risk of RTIs by increasing antimicrobial peptide gene expression, thereby increasing serum concentrations of human cathelicidin (LL-37) and defensins [52]; it also shifts the cytokine balance from proinflammatory T-helper (Th1) cell cytokine production to the production of anti-inflammatory Th2 cell cytokines [132].

Interest in vitamin D’s role in RTIs began before the antibiotic era with a finding of reduced tuberculous illness with exposure to sunshine. That interest reignited with publication of the hypothesis that seasonal variations in solar UVB doses might explain seasonal variations in epidemic influenza, with peak rates in winter, from Cannell and colleagues [133]. That hypothesis was later shown to be only partially correct since temperature and absolute humidity also play important roles [134], while UV has a minor role [135]. However, that hypothesis did lead to further studies, e.g., showing that vitamin D supplementation reduced the risk of influenza A but not influenza B [136]. A meta-analysis of vitamin D RCTs for acute RTIs showed that daily or weekly supplementation with vitamin D significantly reduced risk of acute RTIs [6]. A later meta-analysis reported that maximal protection from vitamin D supplementation was achieved with daily doses of <400 IU for up to 12 months in younger participants (aged 1–16 years) with baseline 25(OH)D concentrations < 10 ng/mL [25]. 

#### 2.1.9. Alzheimer’s Disease and Other Dementias

The mechanisms by which vitamin D reduces risk of AD and dementia are reasonably well understood. Vitamin D triggers several neural pathways that may protect against those neurodegenerative mechanisms, including the deposition of amyloid plaques, inflammatory processes, neurofibrillary degeneration, glutamatergic excitotoxicity, excessive intraneuronal calcium influx, and oxidative stress [137].

**Figure 3:**
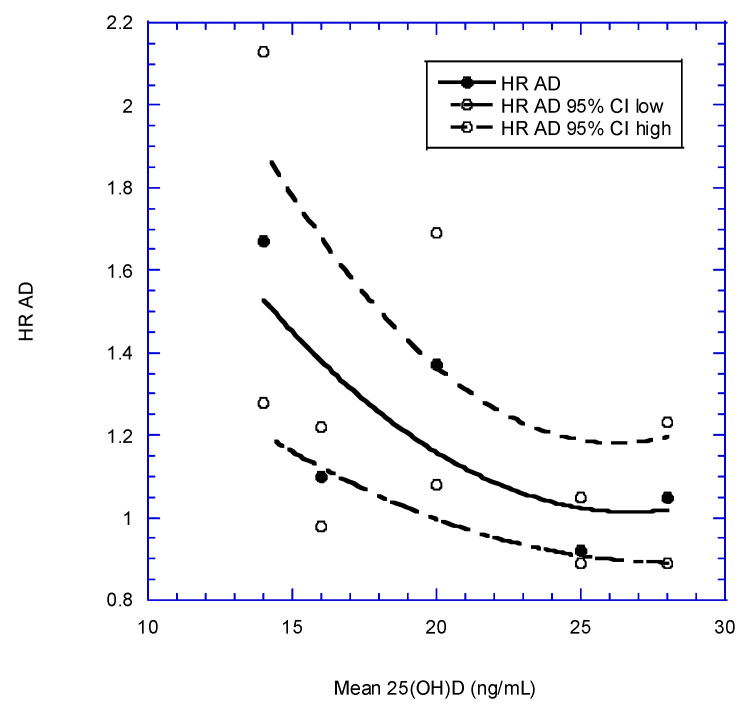
Hazard ratios (HR; mean and 95% confidence interval [CI]) for Alzheimer’s disease (AD) according to mean 25-hydroxyvitamin D [25(OH)D] concentrations for each of the six studies included in the meta-analysis [138].

Two recent meta-analyses looked at dementia and AD risks [138,139]. Jayedi and colleagues calculated the reduction in risk of dementia or AD for a 10-ng/mL increase in 25(OH)D for each study. Table 3 presents the data used in their meta-analysis. Values for mean 25(OH)D concentration were obtained from each article (except Littlejohns et al.). Mean 25(OH)D concentration was taken as the midpoint of 25(OH)D associated with a change in the HR in their Figure 2 and they found that higher 25(OH)D concentrations (up to 32 ng/mL) significantly reduced risk of dementia when Swedish study data [140] were omitted. Figure 2 shows the results for dementia (regression fit equation; HR = 3.3 − 0.16 × [25(OH)D] + 0.0027 × [25(OH)D]^2^, r = 0.95). Figure 3 shows the same equation for AD; HR = 3.3 − 0.17 × [25(OH)D] + 0.0032 × [25(OH)D]^2^, r = 0.75. This analysis highlights the value of considering different ways of doing meta-analyses of observational study data with vitamin D status. Graphs for follow-up time were also generated, but proved less informative than those based on 25(OH)D concentrations.

MR studies [147,148,149] have reported genetically predicted 25(OH)D concentrations correlating inversely with AD risk, as do vitamin D protein binding levels [150] with dementia risks [39].

#### 2.1.10. Major Depressive Disorder

Neuroinflammation appears to be the key factor in onset and progression of MDD [151]. Mechanisms likely to underlie the beneficial effects reported for vitamin D in treating MDD [152] reviewed were, in particular, its antioxidant, anti-inflammatory, proneurogenic, and neuromodulatory properties. Vitamin D also modulates concentrations of gut microbiota, reducing bacterially induced activation of NF-κB in the intestine, thereby reducing remote inflammation further [153].

A meta-analysis of 29 RCTs with 4504 participants concluded that vitamin D supplementation reduced MDD incidence (standardized mean difference: −0.23) and improved responses to the treatment of depression (standardized mean difference: −0.92) [154]. The effects of 2800 IU/d vitamin D over >8 weeks were significant for both prevention and treatment. A meta-analysis of data from seven prospective observational studies of 16,287 older adults with 1157 cases of incident depression reported a pooled HR for depression per 10-ng/mL increase in 25(OH)D of 0.88 (95% CI, 0. 78–0.99) [155].

Another meta-analysis including 41 RCTs with 53,235 participants reported that vitamin D supplementation alleviated depressive symptoms (Hedges’s *g* = −0.32 [95% CI, −0.41 to −0.23]; *p* < 0.001; *I*^2^ = 88%; grade, very low certainty) [156]. Vitamin D supplementation > 2000 IU/d reduced depressive symptoms (*g* = −0.41 [95% CI, −0.56 to −0.26]) much better than <2000 IU/d (*g* = −0.18 [95% CI, −0.29 to −0.08]). A ~linear decrease in HR was seen with increases in serum 25(OH)D concentration, HR falling to 0.43 (95% CI, 0.20–0.92) at 65 ng/mL.

A nested case–control study conducted in Taiwan indicated that although moderate UVB exposure reduced risk of depression, high UVB exposure increased this risk [157]. The adjusted incidence rate ratio for moderate versus low UVB exposure was 0.89 (95% CI, 0.84–0.95); for high UVB, 1.12 (95% CI, 1.02–1.26); for very high UVB, 1.71 (95% CI, 1.51–1.95); and for extreme UVB, 2.79 (95% CI, 2.44–3.18). The authors proposed that high UVB exposure increased production of reactive oxygen species, thereby increasing inflammation. Those results also suggested that observational studies of risk of depression with changes in serum 25(OH)D concentration might be affected by whether 25(OH)D concentrations are raised by solar UVB exposure or by vitamin D supplementation in participants with higher rather than deficient baseline 25(OH)D concentrations.

A study based on the UK Biobank and two other databases showed that serum 25(OH)D concentrations were lower in participants with depression. However, MR analysis reported nonsignificant correlations between genetically determined 25(OH)D concentrations and risk of depression [158]. Thus, depression may lower 25(OH)D concentrations, perhaps by reducing sunlight exposure.

#### 2.1.11. Pregnancy Disorders and Neonatal Outcomes

Vitamin D status during pregnancy is important for both mother and fetus. Wagner and Hollis published a comprehensive review on this topic in mid-2018 [159]. Observational studies reported significant inverse correlations between maternal 25(OH)D concentrations and risk of maternal problems, including preeclampsia [160], altered placental vascular pathology [161], cesarean delivery rates [162,163] gestational diabetes [164,165], and preterm birth rates [166,167]. Such studies also report similar associations for infant health outcomes, including brain dysfunction [168,169] and respiratory disorders [170]. Table 4 shows representative pregnancy outcomes with respect to maternal serum 25(OH)D concentrations from several observational studies [165,166,167,168,169,170,171,172,173,174,175,176,177,178,179,180].

##### Pregnancy Outcomes in Interventional Studies

An open-label vitamin D supplementation observational study involved 1064 women delivering singleton births at the Medical University of South Carolina between September 2015 and December 2016 [14]. Women were given free bottles of 5000-IU vitamin D_3_ and counseled on how to achieve a 25(OH)D concentration >40 ng/mL. Achieved 25(OH)D concentration was also measured. The goal was to see whether raising serum 25(OH)D concentration >40 ng/mL could reduce risk of preterm birth (<37 weeks of gestation). The socioeconomic status aOR was 0.41 (95% CI, 0.24–0.72). The gestation period increased from 36.8 ± 0.3 weeks with 25(OH)Ds~5 ng/mL to 38.3 ± 0.2 weeks at 25 ng/mL after supplementation

Meta-analyses of RCTs have largely, but not consistently, reported benefits of vitamin D supplementation in reducing risks of adverse pregnancy outcomes including low birth weight [173,174], cesarean delivery rates [175], gestational diabetes [174], preeclampsia [176], and preterm delivery [177] (see Table 5).

Maternal vitamin D deficiency leads to epigenetic changes in offspring and is associated with increased risks to bone health in childhood and to increased childhood obesity, those problems appearing to persist into later life. These changes serve as a further reason to ensure vitamin D adequacy in pregnancy [178,179].

## 3. Discussion

Why most vitamin D RCTs used small vitamin D doses—generally at or below 2000 IU/d—is puzzling. One reason may be the Institute of Medicine’s 2011 report setting 4000 IU/d of vitamin D as the upper limit and recommending 600 IU/d for those aged up to 70 years and 800 IU/d for those aged above 70 years—intakes aiming to achieve 25(OH)D of at least 20 ng/mL [180]. The committee was concerned about reports of U-shaped 25(OH)D concentration–health outcome relationships at that time because of a National Cancer Institute review [181] of findings from prospective studies with respect to baseline 25(OH)D concentrations for breast, esophageal, pancreatic, and prostate cancer. The cited studies had drawbacks such as long follow-up times up to 15 years, which reduce the apparent benefit of higher baseline 25(OH)D concentration [16]; not evaluating whether participants changed vitamin D supplementation practices before or during the follow-up periods; and not evaluating vitamin D status during the study or at study completion. Some U-shaped 25(OH)D concentration–health outcome relationships seen have been proposed to be due to participants’ starting vitamin D supplementation shortly before enrolling in prospective studies, for example, after recommendations by doctors over concerns for bone health [56]. Meta-analyses of prospective studies now report inverse relationships with 25(OH)D concentrations for breast cancer incidence from case–control and nested case–control studies, nonsignificant relationships for pancreatic cancer incidence, and direct relationships for prostate cancer incidence [16]. Prostate cancer is unique in that increased risk of mild prostate cancer is due to increased absorption of calcium and phosphorus [16]. However, a number of observational studies have reported U-shaped or reversed J-shaped relations for mortality rates with respect to serum 25(OH)D concentrations such as one that found a significantly increased risk of mortality rate for 25(OH)D concentrations > 120 ng/mL [182].

Several recent vitamin D supplementation observational studies showed that higher daily-dose vitamin D_3_ supplementation was relatively safe and effective. One such study involving 19 lactating women reported that 6400 IU/d of vitamin D_3_ supplementation safely raised 25(OH)D concentrations from 32 ± 4 to 59 ± 7 ng/mL [183]. That group of researchers provided concrete evidence about the safety of 6400 IU/d even for pregnant women to the NIH. A study in Canada had several thousand participants taking vitamin D_3_ doses of their choice. A total of 2229 participants achieved a 25(OH)D concentration > 40 ng/mL by taking 2600 ± 2800 IU/d (for those with mean (SD) value of 47 ± 5 ng/mL) to 6300 ± 500 IU/d, for a mean (SD) value of 117 ± 15 ng/mL [12].

More recently, McCullough and colleagues [57] reported that supplementing over 400 hospital inpatients with 5000–50,000 IU/d of vitamin D_3_ for 30 months was safe and significantly raised serum 25(OH)D concentrations and lowered PTH concentrations. Thus, future vitamin D RCTs should use higher doses of vitamin D_3_ where necessary, for example, to achieve target status. However, although we have solid safety data on vitamin D supplementation, we should nevertheless be cautious, particularly with using high doses in older and/or ill people who may be more prone to vitamin D toxicity.

**Hill’s criteria for causality in a biological system.**

Long before vitamin D RCTs were conducted, Sir Austin Bradford Hill outlined the criteria for causality in a biological system in an address to the Royal Society of Medicine in 1965 [184]. The criteria important for vitamin D include:

1. Strength of association
2. Consistency in findings
3. Temporality, that is, the risk factor must be experienced before the event
4. Biological gradient, that is, dose–response relationship.
5. Plausibility, for example, mechanisms that can explain the relationship
6. Coherence with known biological facts
7. Experiment, for example, RCT
8. Analogy with related associations

Added later [185]:

9. Accounting for confounding factors
10. Accounting for bias such as publication bias
11. Quality of study design

Hill stated that not all criteria need be satisfied to claim causality, but the more that are, the better.

Temporality seems to be interpreted as meaning that prospective studies after measurement of 25(OH)D should be used; however, as discussed, that approach can lead to underestimating the effect of higher 25(OH)D concentration. As long as the disease state does not affect serum 25(OH)D concentration, case–control studies with 25(OH)D measured near time of diagnosis should be acceptable.

Hill’s criteria have been evaluated for vitamin D’s role in several diseases, mainly using observational studies. Those studies reported beneficial effects of vitamin D on the basis of serum 25(OH)D concentrations, finding that nearly all criteria are satisfied except, in some cases, experimental verification for BP [12], cancer [186,187,188,189], CVD [190,191], COVID-19 [94,192], dementia [193], diabetes and pancreatic cancer [194], type 1 diabetes [195], MS [196,197], oral health [198], and periodontal disease [199].

## 4. Conclusions

Observational studies consistently report significant inverse correlations between serum 25(OH)D concentrations and health outcomes, but residual confounding cannot be completely excluded. Prospective studies are preferred over case–control studies on the basis of concern that the disease state could affect serum 25(OH)D concentrations. However, that concern appears to be mainly relevant for inflammatory and infectious diseases such as acute respiratory infections and not other outcomes. The same concern, however, exists when diseases are diagnosed at an advanced stage in their development, as with cancer. One major problem with prospective observational studies is that serum 25(OH)D concentrations change not only with season but also with respect to time, hence resulting in a potential underestimation of the effect of higher 25(OH)D concentrations as discussed earlier.

Another issue regarding observational studies is whether the cases and controls are well matched. Propensity score matching can help ensure good matches, given a sufficiently large pool of prospective controls. Another less-recognized limitation is that if cases and controls are not matched closely with respect to when blood was drawn to measure 25(OH)D concentration, additional bias may be introduced.

Another concern is whether all pertinent confounding factors were considered in the study’s design and analysis. For causality, it is proposed that results of observational studies be included in the broader context of what is known about vitamin D in the health outcome of interest—for example, in relation to mechanistic data or on evaluation using Hill’s criteria for causality [184].

RCTs have largely failed to support vitamin D’s role in reducing risk of adverse health outcomes [15]. The main reasons appear to be that few vitamin D–deficient participants are enrolled, that low vitamin D doses are used, and that outcomes are evaluated by vitamin D dose rather than by achieved 25(OH)D concentrations. Moreover, secondary analyses not proposed in the trial protocols are generally ignored. Those analyses seem to be disregarded based on the concern that if many secondary analyses are conducted, some might accidentally find a significant result, in particular if multiple testing issues are not adequately considered. However, if the secondary outcome is one that researchers forgot to include in the protocol but which reasonably makes sense to include based on other evidence or on mechanistic data, such as the effect of race/ethnicity and BMI on cancer risk in the VITAL study [8]—then such evidence should rather be accepted. Given the time, effort and expense required for major vitamin D RCTs, it seems unlikely that many more will be conducted soon, or ever. Thus, it is imperative to learn to use findings from completed trials and from other research approaches as efficiently as possible.

Examining the role of genetic factors might also be helpful in understanding the inconsistency of results between observational studies and RCTs since supplements might interact differently according to specific genotypes and variants [200]. Unfortunately, such potential genotype/supplement interactions have rarely been examined in RCT settings.

Observational studies using variable vitamin D dose supplementation and frequent (annual or semiannual) measurement of 25(OH)D concentrations and other pertinent data have several apparent advantages over traditional observational studies and RCTs. Permitting variable vitamin D doses allows large 25(OH)D ranges to be covered, which should make any significant health benefits more likely to become apparent [11,12].

MR studies that establish vitamin D’s causal role for several health outcomes are now being reported. By using genetically predicted 25(OH)D concentrations for many participants combined with reported health outcomes, MR studies of large cohorts average out lifestyle factors in ways that match the effects of randomization in RCTs. Recently, stratifying subjects into ~100 subgroups by baseline 25(OH)D concentrations for separate MR analyses (which allows analysis of nonlinear data) provides an excellent approach to examining the effects of genetic increases in serum 25(OH)D at low baseline 25(OH)D concentrations [40], especially when those effects cannot be adequately considered when all the data are included in a single analysis.

Ecological studies have historically been useful in highlighting that UVB radiation reduces risk of diseases such as MS and cancers [42]. The advantages of ecological studies include the fact that many participants are studied and that data for many population-level confounding factors can be used. Geographical ecological studies surpass temporal ecological studies because the multiple factors involved in seasonal variations are hard to untangle, including UVB production of vitamin D, UVB non–vitamin D mechanisms, UVA-induced increases in serum NO concentrations, and temperature [45].

Knowledge of the mechanisms whereby vitamin D reduces particular adverse health outcomes is fundamental to understanding the problems of deficiency and should be routinely considered in designing future trials. The mechanisms by which correcting deficiencies can reduce tissue dysfunction in each of the common disorders we have discussed are all reasonably well understood which supports the case for ensuring better vitamin D provision in populations commonly afflicted with deficiency.

## Figures and Tables

**Table 1:** Findings regarding cancer incidence and mortality rates with respect to 25(OH)D concentrations or vitamin D supplementation.

Study	Change in 25(OH)D	Incidence, RR or HR (95% CI)	Mortality, RR or HR (95% CI)	Author
Observational Studies				
Harvard Health Professionals Follow-Up Study, all cancer	10 ng/mL	RR = 0.83 (0.74–0.92)	RR = 0.71 (0.60–0.83)	Giovannucci et al., [65]
Adult patients living in Olmsted County, Minnesota, all less skin cancer	<12 ng/mL	HR = 1.56 (1.03–2.36)	HR = 2.35 (1.01–5.48)	Johnson et al., [66]
Meta-analysis, breast cancer	High vs. low	RR = 0.92 (0.83–1.02)	RR = 0.58 (0.40–0.85)	Kim et al., [67]
VITAL, exclude first 2 years	Vitamin D treatment vs. placebo	HR = 0.94 (0.83–1.06)	HR = 0.75 (0.59–0.96)	Manson et al., [8]
**RCTs**				
RCTs, meta-analysis	All participants	(12 RCTs) SRR = 0.99 (0.04–1.03)	(6 RCT) SRR = 0.92 (0.82–1.03)	Keum et al., [24]
RCTs, meta-analysis	Normal-weight individuals	(1 RCT) SRR = 0.76 (0.60–0.94)		Keum et al., [24]

Abbreviations: 25(OH)D, 25-hydroxyvitamin D; 95% CI, 95% confidence interval; HR, hazard ratio; RCT, randomized controlled trial; RR, relative risk; SRR, summary RR; VITAL, VITamin D and OmegA-3 TriaL.

**Table 2:** Data on mean 25(OH)D concentration and aHR for CVD mortality extracted from four research articles as further adjusted as just discussed [78,79,80,81].

Mean 25(OH)D Concentration (ng/mL)	aHR	aHR Adjusted	aHR Adjusted, 95% CI Low	aHR Adjusted, 95% CI High	Author
12.5	1.20	1.39	1.01	1.90	Melamed et al., [79]
21.0	0.88	1.02	0.80	1.32	
28.3	0.83	0.96	0.75	1.24	
36.0	1.00	1.16	1.16	1.16	
7.00		2.36	1.17	4.75	Ginde et al. [78]
15.0		1.54	1.01	2.34	
25.0		1.26	0.85	1.88	
35.0		1.20	0.79	1.81	
45.0		1.00	1.00	1.00	
7.00	2.64	3.55	2.27	2.96	Semba et al., [80]
13.0	1.68	2.26	1.03	5.02	
21.0	2.19	2.95	1.42	6.20	
30.0	1.00	1.35	1.35	1.35	
15.0	1.52	1.79	1.52	2.11	Liu et al., [81]
25.0	1.09	1.29	1.11	1.51	
35.0	1.00	1.18	1.18	1.18	

Abbreviations: 95% CI, 95% confidence interval; 25(OH)D, 25-hydroxyvitamin D; aHR, adjusted hazard ratio.

**Table 3:** Data associated with the observational studies in the meta-analysis by Jayedi and colleagues [138].

Country	Mean 25(OH)D (ng/mL)	Follow-Up (yrs)	Vascular Dementia, HR (95% CI) for 10 ng/mL Increase	Alzheimer’s, HR (95% CI) for 10 ng/mL Increase	Author
US	12	5.6	0.57 (0.34–0.97)	0.61 (0.41–0.93)	Littlejohns et al., [141]
France	14	11.4	0.60 (0.47–0.78)	0.60 (0.47–0.78)	Feart et al., [142]
Finland	16	17.0	0.77 (0.62–0.92)		Knekt et al., [143]
Denmark	16	21.0		0.91 (0.82–1.02)	Afzal et al., [130]
Netherlands	20	13.3	0.77 (0.63–0.95)	0.73 (0.59–0.93)	Licher et al., [144]
US	22	16.6	0.93 (0.79–1.07)		Schneider et al., [145]
US	25	9.0	1.01 (0.88–1.14)	1.09 (0.95–1.12)	Karakis et al., [146]
Sweden	28	12.0	1.04 (0.93–1.17)	0.95 (0.81–1.12)	Olsson et al., [140]

Abbreviations: 95% CI, 95% confidence interval; 25(OH)D, 25-hydroxyvitamin D; HR, hazard ratio.

**Table 4:** Representative pregnancy and infant outcomes with respect to maternal serum 25(OH)D concentration in observational studies.

Outcome	Setting	Outcome	Finding	Author
Birth weight				
Cesarean delivery, primary		Maternal 25(OH)D < 15 vs. >15 ng/mL	aOR = 3.8 (95% CI, 1.7–8.6)	Merewood et al., [162]
Cesarean delivery, primary		Maternal 25(OH)D < 15 vs. >15 ng/mL	aOR = 2.0 (95% CI, 1.2–3.3)	Scholl et al., [163]
Gestational diabetes	Meta-analysis, 29 studies	<20 vs. >20 ng/mL	OR = 1.39 (95% CI, 1.20–1.60)	Hu et al., [164]
Gestational diabetes	Meta-analysis, 27 studies	>20 vs. >30 ng/mL	OR = 1.26 (95% CI, 1.13–1.41)	Milajerdi et al., [165]
Preeclampsia	Hospital study	Early-onset severe preeclampsia, 10-ng/mL increase in 25(OH)D	aOR = 0.37 (95% CI, 0.22–0.62)	Robinson et al., [171]
Preeclampsia	Meta-analysis, 13 studies	Comparison of 25(OH)D	OR = 0.57 (95% CI, 0.51–0.65)	Serrano-Diaz et al., [172]
Preeclampsia	Meta-analysis, 11 studies	25(OH)D < 30 vs. >30 ng/mL	OR = 1.44 (95% CI, 1.26–1.64)	Aguilar-Cordero et al., [160]
Preterm delivery	Hospital study	25(OH)D < 20 vs. >40 ng/mL, <16 wks	OR = 3.8 (95% CI, 1.4–10.7)	Wagner et al., [166]
Preterm delivery	Meta-analysis, 16 studies	25(OH)D < 20 vs. >20 ng/mL	OR = 1.25 (95% CI, 1.13–1.38)	Zhou et al., [167]
Preterm delivery	Open-label vitamin D supplementation	25(OH)D > 40 vs. >20 ng/mL	SES adjusted OR = 0.41 (95% CI, 0.24–0.72)	McDonnell et al., [14]
Infant outcomes				
Brain dysfunction	Language impairment in childhood vs. maternal 25(OH)D at 18 weeks pregnancy	6-18 vs. 29-62 ng/mL	aOR = 1.97 (95% CI, 1.00–3.93, *p* < 0.05)	Whitehouse et al., [168]
Brain dysfunction	Risk of ADHD, meta-analysis, 5 studies	High vs. low 25(OH)D	OR/RR = 0.72 (95% CI, 0.59–0.89)	Garcia-Serna et al., [169]
Respiratory dysfunction	Risk of asthma vs. maternal 25(OH)D, 11 studies	High vs. low 25(OH)D	OR = 0.78 (95% CI, 0.69–0.89)	Shi et al., [170]
Respiratory dysfunction	Risk of wheeze vs. maternal 25(OH)D, 14 studies	High vs. low 25(OH)D	OR = 0.65 (95% CI, 0.54–0.79)	Shi et al., [170]

Abbreviations: 25(OH)D, 25-hydroxyvitamin D; 95% CI, 95% confidence interval; ADHD, attention deficit–hyperactivity disorder; aOR, adjusted odds ratio; OR, odds ratio; SES socioeconomic status.

**Table 5:** Meta-analyses of vitamin D supplementation RCTs, comparing vitamin D supplementation with placebo.

Outcome	Setting	Finding	Author
Birth weight, low	Review of 5 RCTs	RR = 0.55 (95% CI, 0.35–0.87)	Palacios et al., [174]
Birth weight	Review of 11 RCTs	Increased weight, mean difference = 114 g (95% CI, 63–165 g)	Gallo et al., [173]
Cesarean delivery, primary	Review of 6 RCTs	OR = 0.9 (95% CI, 0.7–1.2)	Gallo et al., [173]
Cesarean delivery in Iran	Review of 5 RCTs	RR = 0.61 (95% CI, 0.44–0.83)	Saha and Saha, [175]
Gestational diabetes	Review of 4 RCTs	RR = 0.51 (95% CI, 0.27–0.97)	Palacios et al., [174]
Preeclampsia	Review of 27 RCTs	RR = 0.37 (95% CI, 0.26–0.52)	Fogacci et al., [176]
Preterm delivery	Review of 17 RCTs	RR = 0.70 (95% CI, 0.49–1.00)	Kinshella et al., [177]

Abbreviations: 95% CI, 95% confidence interval; OR, odds ratio; RCT, randomized controlled trial; RR, relative risk.

## Data Availability

Not applicable.

## References

[1] Jones G. (2022). 100 YEARS OF VITAMIN D: Historical aspects of vitamin D. Endocr. Connect..

[2] Autier P., Boniol M., Pizot C., Mullie P. (2014). Vitamin D status and ill health: A systematic review. Lancet Diabetes Endocrinol..

[3] Adams J.S., Rafison B., Witzel S., Reyes R.E., Shieh A., Chun R., Zavala K., Hewison M., Liu P.T. (2014). Regulation of the extrarenal CYP27B1-hydroxylase. J. Steroid Biochem. Mol. Biol..

[4] Autier P., Mullie P., Macacu A., Dragomir M., Boniol M., Coppens K., Pizot C., Boniol M. (2017). Effect of vitamin D supplementation on non-skeletal disorders: A systematic review of meta-analyses and randomised trials. Lancet Diabetes Endocrinol..

[5] Rejnmark L., Bislev L.S., Cashman K.D., Eiríksdottir G., Gaksch M., Grüebler M., Grimnes G., Gudnason V., Lips P., Pilz S. (2017). Non-skeletal health effects of vitamin D supplementation: A systematic review on findings from meta-analyses summarizing trial data. PLoS ONE.

[6] Martineau A.R., Jolliffe D.A., Hooper R.L., Greenberg L., Aloia J.F., Bergman P., Dubnov-Raz G., Esposito S., Ganmaa D., Ginde A.A. (2017). Vitamin D supplementation to prevent acute respiratory tract infections: Systematic review and meta-analysis of individual participant data. BMJ.

[7] Maretzke F., Bechthold A., Egert S., Ernst J.B., Melo van Lent D., Pilz S., Reichrath J., Stangl G.I., Stehle P., Volkert D. (2020). Role of Vitamin D in Preventing and Treating Selected Extraskeletal Diseases—An Umbrella Review. Nutrients.

[8] Manson J.E., Cook N.R., Lee I.M., Christen W., Bassuk S.S., Mora S., Gibson H., Gordon D., Copeland T., D’Agostino D. (2019). Vitamin D Supplements and Prevention of Cancer and Cardiovascular Disease. N. Engl. J. Med..

[9] Pittas A.G., Dawson-Hughes B., Sheehan P., Ware J.H., Knowler W.C., Aroda V.R., Brodsky I., Ceglia L., Chadha C., Chatterjee R. (2019). Vitamin D Supplementation and Prevention of Type 2 Diabetes. N. Engl. J. Med..

[10] Smolders J., van den Ouweland J., Geven C., Pickkers P., Kox M. (2021). Letter to the Editor: Vitamin D deficiency in COVID-19: Mixing up cause and consequence. Metabolism.

[11] McDonnell S.L., Baggerly C.A., French C.B., Baggerly L.L., Garland C.F., Gorham E.D., Hollis B.W., Trump D.L., Lappe J.M. (2018). Breast cancer risk markedly lower with serum 25-hydroxyvitamin D concentrations ≥60 vs. <20 ng/mL (150 vs. 50 nmol/L): Pooled analysis of two randomized trials and a prospective cohort. PLoS ONE.

[12] Mirhosseini N., Vatanparast H., Kimball S.M. (2017). The Association between Serum 25(OH)D Status and Blood Pressure in Participants of a Community-Based Program Taking Vitamin D Supplements. Nutrients.

[13] Acharya P., Dalia T., Ranka S., Sethi P., Oni O.A., Safarova M.S., Parashara D., Gupta K., Barua R.S. (2021). The Effects of Vitamin D Supplementation and 25-Hydroxyvitamin D Levels on the Risk of Myocardial Infarction and Mortality. J. Endocr. Soc..

[14] McDonnell S.L., Baggerly K.A., Baggerly C.A., Aliano J.L., French C.B., Baggerly L.L., Ebeling M.D., Rittenberg C.S., Goodier C.G., Mateus Nino J.F. (2017). Maternal 25(OH)D concentrations ≥40 ng/mL associated with 60% lower preterm birth risk among general obstetrical patients at an urban medical center. PLoS ONE.

[15] Pilz S., Trummer C., Theiler-Schwetz V., Grübler M.R., Verheyen N.D., Odler B., Karras S.N., Zittermann A., März W. (2022). Critical Appraisal of Large Vitamin D Randomized Controlled Trials. Nutrients.

[16] Muñoz A., Grant W.B. (2022). Vitamin D and Cancer: An Historical Overview of the Epidemiology and Mechanisms. Nutrients.

[17] Garland C., Barrett-Connor E., Rossof A., Shekelle R., Criqui M., Paul O. (1985). Dietary Vitamin D and Calcium and Risk of Colorectal Cancer: A 19-Year Prospective Study in Men. Lancet.

[18] O’Neill C.M., Kazantzidis A., Kiely M., Cox L., Meadows S., Goldberg G., Prentice A., Kift R., Webb A.R., Cashman K.D. (2017). A predictive model of serum 25-hydroxyvitamin D in UK white as well as black and Asian minority ethnic population groups for application in food fortification strategy development towards vitamin D deficiency prevention. J. Steroid Biochem. Mol. Biol..

[19] Crowe F.L., Steur M., Allen N.E., Appleby P.N., Travis R.C., Key T.J. (2011). Plasma concentrations of 25-hydroxyvitamin D in meat eaters, fish eaters, vegetarians and vegans: Results from the EPIC–Oxford study. Public Health Nutr..

[20] Scragg R. (2018). Limitations of vitamin D supplementation trials: Why observational studies will continue to help determine the role of vitamin D in health. J. Steroid Biochem. Mol. Biol..

[21] Boucher B.J., Grant W.B. (2019). Re: Scragg–Emerging Evidence of Thresholds for Beneficial Effects from Vitamin D Supplementation. Nutrients.

[22] Hujoel P.P. (2013). Vitamin D and dental caries in controlled clinical trials: Systematic review and meta-analysis. Nutr. Rev..

[23] Christiansen C., Rødbro P., Lund M. (1973). Effect of Vitamin D on Bone Mineral Mass in Normal Subjects and in Epileptic Patients on Anticonvulsants: A Controlled Therapeutic Trial. BMJ.

[24] Keum N., Chen Q.-Y., Lee D.H., Manson J.E., Giovannucci E. (2022). Vitamin D supplementation and total cancer incidence and mortality by daily vs. infrequent large-bolus dosing strategies: A meta-analysis of randomised controlled trials. Br. J. Cancer.

[25] Jolliffe D.A., Camargo C.A., Sluyter J.D., Aglipay M., Aloia J.F., Ganmaa D., Bergman P., Bischoff-Ferrari H.A., Borzutzky A., Damsgaard C.T. (2021). Vitamin D supplementation to prevent acute respiratory infections: A systematic review and meta-analysis of aggregate data from randomised controlled trials. Lancet Diabetes Endocrinol..

[26] Hahn J., Cook N.R., Alexander E.K., Friedman S., Walter J., Bubes V., Kotler G., Lee I.-M., E Manson J., Costenbader K.H. (2022). Vitamin D and marine omega 3 fatty acid supplementation and incident autoimmune disease: VITAL randomized controlled trial. BMJ.

[27] Heaney R.P. (2014). Guidelines for optimizing design and analysis of clinical studies of nutrient effects. Nutr. Rev..

[28] Grant W.B., Boucher B.J., Bhattoa H.P., Lahore H. (2018). Why vitamin D clinical trials should be based on 25-hydroxyvitamin D concentrations. J. Steroid Biochem. Mol. Biol..

[29] Lelieveld J., Klingmüller K., Pozzer A., Pöschl U., Fnais M., Daiber A., Münzel T. (2019). Cardiovascular disease burden from ambient air pollution in Europe reassessed using novel hazard ratio functions. Eur. Heart J..

[30] DiNicolantonio J.J., O’Keefe J.H., Wilson W. (2018). Subclinical magnesium deficiency: A principal driver of cardiovascular disease and a public health crisis. Open Heart.

[31] Lappe J.M., Travers-Gustafson D., Davies K.M., Recker R.R., Heaney R.P. (2007). Vitamin D and calcium supplementation reduces cancer risk: Results of a randomized trial. Am. J. Clin. Nutr..

[32] Reid I.R., Birstow S.M., Bolland M. (2017). Calcium and Cardiovascular Disease. Endocrinol. Metab..

[33] Wang T.J., Zhang F., Richards J.B., Kestenbaum B., van Meurs J.B., Berry D., Kiel D.P., Streeten E.A., Ohlsson C., Koller D.L. (2010). Common genetic determinants of vitamin D insufficiency: A genome-wide association study. Lancet.

[34] Hatchwell E., Greally J.M. (2007). The potential role of epigenomic dysregulation in complex human disease. Trends Genet..

[35] Kilpinen H., Dermitzakis E.T. (2012). Genetic and epigenetic contribution to complex traits. Hum. Mol. Genet..

[36] Wong A.K., Sealfon R.S.G., Theesfeld C.L., Troyanskaya O.G. (2021). Decoding disease: From genomes to networks to phenotypes. Nat. Rev. Genet..

[37] Revez J.A., Lin T., Qiao Z., Xue A., Holtz Y., Zhu Z., Zeng J., Wang H., Sidorenko J., Kemper K.E. (2020). Genome-wide association study identifies 143 loci associated with 25 hydroxyvitamin D concentration. Nat. Commun..

[38] Ong J.S., Dixon-Suen S.C., Han X., An J., Liyanage U., Dusingize J.C., Schumacher J., Gockel I., Esophageal Cancer Consortium, 23 and Me Research Team (2021). A comprehensive re-assessment of the association between vitamin D and cancer susceptibility using Mendelian randomization. Nat. Commun..

[39] Navale S.S., Mulugeta A., Zhou A., Llewellyn D.J., Hyppönen E. (2022). Vitamin D and brain health: An observational and Mendelian randomization study. Am. J. Clin. Nutr..

[40] Zhou A., Selvanayagam J.B., Hyppönen E. (2022). Non-linear Mendelian randomization analyses support a role for vitamin D deficiency in cardiovascular disease risk. Eur. Heart J..

[41] Garland C.F., Garland F.C. (1980). Do sunlight and vitamin D reduce the likelihood of colon cancer?. Int. J. Epidemiol..

[42] Grant W.B. (2016). The role of geographical ecological studies in identifying diseases linked to UVB exposure and/or vitamin D. Derm.-Endocrinol..

[43] Grant W.B. (2002). An estimate of premature cancer mortality in the U.S. due to inadequate doses of solar ultraviolet-B radiation. Cancer.

[44] Grant W.B., Garland C.F. (2006). The association of solar ultraviolet B (UVB) with reducing risk of cancer: Multifactorial ecologic analysis of geographic variation in age-adjusted cancer mortality rates. Anticancer Res..

[45] Grant W.B., Boucher B.J. (2022). An Exploration of How Solar Radiation Affects the Seasonal Variation of Human Mortality Rates and the Seasonal Variation in Some Other Common Disorders. Nutrients.

[46] Doll R., Peto R., Boreham J., Sutherland I. (2005). Mortality from cancer in relation to smoking: 50 years observations on British doctors. Br. J. Cancer.

[47] Murdaca G., Tonacci A., Negrini S., Greco M., Borro M., Puppo F., Gangemi S. (2019). Emerging role of vitamin D in autoimmune diseases: An update on evidence and therapeutic implications. Autoimmun. Rev..

[48] Dankers W., Colin E.M., van Hamburg J.P., Lubberts E. (2017). Vitamin D in Autoimmunity: Molecular Mechanisms and Therapeutic Potential. Front. Immunol..

[49] Prummel M.F., Strieder T., Wiersinga W.M. (2004). The environment and autoimmune thyroid diseases. Eur. J. Endocrinol..

[50] Oliver J.E., Silman A.J. (2006). Risk factors for the development of rheumatoid arthritis. Scand. J. Rheumatol..

[51] Huerta C., Rivero E., Rodríguez L.A.G. (2007). Incidence and Risk Factors for Psoriasis in the General Population. Arch. Dermatol..

[52] Gombart A.F. (2009). The vitamin D–antimicrobial peptide pathway and its role in protection against infection. Future Microbiol..

[53] Guillot X., Semerano L., Saidenberg-Kermanac’h N., Falgarone G., Boissier M.C. (2010). Vitamin D and inflammation. Jt. Bone Spine.

[54] Carlberg C., Muñoz A. (2022). An update on vitamin D signaling and cancer. Semin. Cancer Biol..

[55] Garland C., Garland F.C., Shaw E., Comstock G.W., Helsing K.J., Gorham E.D. (1989). Serum 25-Hydroxyvitamin D and Colon Cancer: Eight-Year Prospective Study. Lancet.

[56] Grant W.B., Karras S.N., Bischoff-Ferrari H.A., Annweiler C., Boucher B.J., Juzeniene A., Garland C.F., Holick M.F. (2016). Do studies reporting ‘U’-shaped serum 25-hydroxyvitamin D-health outcome relationships reflect adverse effects?. Derm.-Endocrinol..

[57] McCullough M.L., Zoltick E.S., Weinstein S.J., Fedirko V., Wang M., Cook N.R., Eliassen A.H., Zeleniuch-Jacquotte A., Agnoli C., Albanes D. (2019). Circulating Vitamin D and Colorectal Cancer Risk: An International Pooling Project of 17 Cohorts. J. Natl. Cancer Inst..

[58] Grant W.B. (2011). Effect of interval between serum draw and follow-up period on relative risk of cancer incidence with respect to 25-hydroxyvitamin D level; implications for meta-analyses and setting vitamin D guidelines. Derm.-Endocrinol..

[59] Grant W.B. (2012). Effect of follow-up time on the relation between prediagnostic serum 25-hydroxyvitamin D and all-cause mortality rate. Derm.-Endocrinol..

[60] Grant W.B. (2015). 25-hydroxyvitamin D and breast cancer, colorectal cancer, and colorectal adenomas: Case-control versus nested case-control studies. Anticancer Res..

[61] Lappe J., Watson P., Travers-Gustafson D., Recker R., Garland C., Gorham E., Baggerly K., McDonnell S.L. (2017). Effect of Vitamin D and Calcium Supplementation on Cancer Incidence in Older Women: A Randomized Clinical Trial. JAMA.

[62] Ekwaru J.P., Zwicker J.D., Holick M.F., Giovannucci E., Veugelers P.J. (2014). The Importance of Body Weight for the Dose Response Relationship of Oral Vitamin D Supplementation and Serum 25-Hydroxyvitamin D in Healthy Volunteers. PLoS ONE.

[63] Wamberg L., Kampmann U., Stodkilde-Jorgensen H., Rejnmark L., Pedersen S.B., Richelsen B. (2013). Effects of vitamin D supplementation on body fat accumulation, inflammation, and metabolic risk factors in obese adults with low vitamin D levels—Results from a randomized trial. Eur. J. Intern. Med..

[64] Michels N., van Aart C., Morisse J., Mullee A., Huybrechts I. (2021). Chronic inflammation towards cancer incidence: A systematic review and meta-analysis of epidemiological studies. Crit. Rev. Oncol..

[65] Giovannucci E., Liu Y., Rimm E.B., Hollis B.W., Fuchs C.S., Stampfer M.J., Willett W.C. (2006). Prospective Study of Predictors of Vitamin D Status and Cancer Incidence and Mortality in Men. J. Natl. Cancer Inst..

[66] Johnson C.R., Dudenkov D.V., Mara K.C., Fischer P.R., Maxson J.A., Thacher T.D. (2021). Serum 25-Hydroxyvitamin D and Subsequent Cancer Incidence and Mortality: A Population-Based Retrospective Cohort Study. Mayo Clin. Proc..

[67] Kim Y., Je Y. (2014). Vitamin D intake, blood 25(OH)D levels, and breast cancer risk or mortality: A meta-analysis. Br. J. Cancer.

[68] Gnagnarella P., Muzio V., Caini S., Raimondi S., Martinoli C., Chiocca S., Miccolo C., Bossi P., Cortinovis D., Chiaradonna F. (2021). Vitamin D Supplementation and Cancer Mortality: Narrative Review of Observational Studies and Clinical Trials. Nutrients.

[69] Li Z., Wu L., Zhang J., Huang X., Thabane L., Li G. (2021). Effect of Vitamin D Supplementation on Risk of Breast Cancer: A Systematic Review and Meta-Analysis of Randomized Controlled Trials. Front. Nutr..

[70] Devesa S.S., Grauman D.J., Blot W.J., Pennelo G.A., Hoover R.N., Fraumeni J.F. (1999). Atlas of Cancer Mortality in the United States, 1950–1994.

[71] Marti-Soler H., Gonseth S., Gubelmann C., Stringhini S., Bovet P., Chen P.-C., Wojtyniak B., Paccaud F., Tsai D.-H., Zdrojewski T. (2014). Seasonal Variation of Overall and Cardiovascular Mortality: A Study in 19 Countries from Different Geographic Locations. PLoS ONE.

[72] Mozos I., Marginean O. (2015). Links between Vitamin D Deficiency and Cardiovascular Diseases. BioMed Res. Int..

[73] Glovaci D., Fan W., Wong N.D. (2019). Epidemiology of Diabetes Mellitus and Cardiovascular Disease. Curr. Cardiol. Rep..

[74] Nguyen J.L., Yang W., Ito K., Matte T.D., Shaman J., Kinney P.L. (2016). Seasonal Influenza Infections and Cardiovascular Disease Mortality. JAMA Cardiol..

[75] Liccardo D., Cannavo A., Spagnuolo G., Ferrara N., Cittadini A., Rengo C., Rengo G. (2019). Periodontal Disease: A Risk Factor for Diabetes and Cardiovascular Disease. Int. J. Mol. Sci..

[76] Wang T.J., Pencina M.J., Booth S.L., Jacques P.F., Ingelsson E., Lanier K., Benjamin E.J., D’Agostino R.B., Wolf M., Vasan R.S. (2008). Vitamin D Deficiency and Risk of Cardiovascular Disease. Circulation.

[77] Zhang R., Li B., Gao X., Tian R., Pan Y., Jiang Y., Gu H., Wang Y., Wang Y., Liu G. (2017). Serum 25-hydroxyvitamin D and the risk of cardiovascular disease: Dose-response meta-analysis of prospective studies. Am. J. Clin. Nutr..

[78] Ginde A.A., Scragg R., Schwartz R.S., Camargo C.A. (2009). Prospective Study of Serum 25-Hydroxyvitamin D Level, Cardiovascular Disease Mortality, and All-Cause Mortality in Older U.S. Adults. J. Am. Geriatr. Soc..

[79] Melamed M.L., Michos E.D., Post W., Astor B. (2008). 25-Hydroxyvitamin D Levels and the Risk of Mortality in the General Population. Arch. Intern. Med..

[80] Semba R.D., Houston D., Bandinelli S., Sun K., Cherubini A., Cappola A.R., Guralnik J.M., Ferrucci L. (2010). Relationship of 25-hydroxyvitamin D with all-cause and cardiovascular disease mortality in older community-dwelling adults. Eur. J. Clin. Nutr..

[81] Liu L., Chen M., Hankins S.R., Nùñez A.E., Watson R.A., Weinstock P.J., Newschaffer C.J., Eisen H.J. (2012). Serum 25-Hydroxyvitamin D Concentration and Mortality from Heart Failure and Cardiovascular Disease, and Premature Mortality from All-Cause in United States Adults. Am. J. Cardiol..

[82] Austin P.C. (2011). An Introduction to Propensity Score Methods for Reducing the Effects of Confounding in Observational Studies. Multivar. Behav. Res..

[83] Anderson J.L., Vanwoerkom R.C., Horne B.D., Bair T.L., May H.T., Lappe D.L., Muhlestein J.B. (2011). Parathyroid hormone, vitamin D, renal dysfunction, and cardiovascular disease: Dependent or independent risk factors?. Am. Heart J..

[84] Kestenbaum B., Katz R., de Boer I., Hoofnagle A., Sarnak M.J., Shlipak M.G., Jenny N.S., Siscovick D.S. (2011). Vitamin D, Parathyroid Hormone, and Cardiovascular Events Among Older Adults. J. Am. Coll. Cardiol..

[85] Chen W.R., Chen Y.D., Shi Y., Yin D.W., Wang H., Sha Y. (2015). Vitamin D, parathyroid hormone and risk factors for coronary artery disease in an elderly Chinese population. J. Cardiovasc. Med..

[86] Wannamethee S.G., Welsh P., Papacosta O., Lennon L., Whincup P.H., Sattar N. (2014). Elevated Parathyroid Hormone, But Not Vitamin D Deficiency, Is Associated with Increased Risk of Heart Failure in Older Men with and without Cardiovascular Disease. Circ. Heart Fail..

[87] Valcour A., Blocki F., Hawkins D.M., Rao S.D. (2012). Effects of Age and Serum 25-OH-Vitamin D on Serum Parathyroid Hormone Levels. J. Clin. Endocrinol. Metab..

[88] Zhang Z., Yang Y., Ng C.Y., Wang D., Wang J., Li G., Liu T. (2016). Meta-analysis of Vitamin D Deficiency and Risk of Atrial Fibrillation. Clin. Cardiol..

[89] Kahn R., Robertson R.M., Smith R., Eddy D. (2008). The Impact of Prevention on Reducing the Burden of Cardiovascular Disease. Diabetes Care.

[90] Farley T.A., Dalal M.A., Mostashari F., Frieden T.R. (2010). Deaths Preventable in the U.S. by Improvements in Use of Clinical Preventive Services. Am. J. Prev. Med..

[91] Timms P.M., Mannan N., Hitman G.A., Noonan K., Mills P.G., Syndercombe-Court D., Aganna E., Price C.P., Boucher B.J. (2002). Circulating MMP9, vitamin D and variation in the TIMP-1 response with VDR genotype: Mechanisms for inflammatory damage in chronic disorders?. QJM.

[92] Rimondi E., Marcuzzi A., Casciano F., Tornese G., Pellati A., Toffoli B., Secchiero P., Melloni E. (2021). Role of vitamin D in the pathogenesis of atheromatosis. Nutr. Metab. Cardiovasc. Dis..

[93] Grant W.B., Lahore H., McDonnell S.L., Baggerly C.A., French C.B., Aliano J.L., Bhattoa H.P. (2020). Evidence that Vitamin D Supplementation Could Reduce Risk of Influenza and COVID-19 Infections and Deaths. Nutrients.

[94] Mercola J., Grant W.B., Wagner C.L. (2020). Evidence Regarding Vitamin D and Risk of COVID-19 and Its Severity. Nutrients.

[95] Kaufman H.W., Niles J.K., Kroll M.H., Bi C., Holick M.F. (2020). SARS-CoV-2 positivity rates associated with circulating 25-hydroxyvitamin D levels. PLoS ONE.

[96] Martineau A.R., Cantorna M.T. (2022). Vitamin D for COVID-19: Where are we now?. Nat. Rev. Immunol..

[97] Oristrell J., Oliva J.C., Casado E., Subirana I., Domínguez D., Toloba A., Balado A., Grau M. (2022). Vitamin D supplementation and COVID-19 risk: A population-based, cohort study. J. Endocrinol. Investig..

[98] Seal K.H., Bertenthal D., Carey E., Grunfeld C., Bikle D.D., Lu C.M. (2022). Association of Vitamin D Status and COVID-19-Related Hospitalization and Mortality. J. Gen. Intern. Med..

[99] Dissanayake H.A., de Silva N.L., Sumanatilleke M., de Silva S.D.N., Gamage K.K.K., Dematapitiya C., Kuruppu D.C., Ranasinghe P., Pathmanathan S., Katulanda P. (2022). Prognostic and Therapeutic Role of Vitamin D in COVID-19: Systematic Review and Meta-analysis. J. Clin. Endocrinol. Metab..

[100] Hosseini B., El Abd A., Ducharme F.M. (2022). Effects of Vitamin D Supplementation on COVID-19 Related Outcomes: A Systematic Review and Meta-Analysis. Nutrients.

[101] De Niet S., Trémège M., Coffiner M., Rousseau A.-F., Calmes D., Frix A.-N., Gester F., Delvaux M., Dive A.-F., Guglielmi E. (2022). Positive Effects of Vitamin D Supplementation in Patients Hospitalized for COVID-19: A Randomized, Double-Blind, Placebo-Controlled Trial. Nutrients.

[102] Norman A.W., Frankel B.J., Heldt A.M., Grodsky G.M. (1980). Vitamin D Deficiency Inhibits Pancreatic Secretion of Insulin. Science.

[103] Hewison M. (2012). Vitamin D and immune function: Autocrine, paracrine or endocrine?. Scand. J. Clin. Lab. Investig. Suppl..

[104] Cheng Q., Boucher B.J., Leung P.S. (2013). Modulation of hypovitaminosis D-induced islet dysfunction and insulin resistance through direct suppression of the pancreatic islet renin–angiotensin system in mice. Diabetologia.

[105] Leung P.S. (2016). The Potential Protective Action of Vitamin D in Hepatic Insulin Resistance and Pancreatic Islet Dysfunction in Type 2 Diabetes Mellitus. Nutrients.

[106] Szymczak-Pajor I., Drzewoski J., Śliwińska A. (2020). The Molecular Mechanisms by Which Vitamin D Prevents Insulin Resistance and Associated Disorders. Int. J. Mol. Sci..

[107] Boucher B.J. (1998). Inadequate vitamin D status: Does it contribute to the disorders comprising syndrome ‘X’?. Br. J. Nutr..

[108] Pittas A.G., Lau J., Hu F.B., Dawson-Hughes B. (2007). The Role of Vitamin D and Calcium in Type 2 Diabetes. A Systematic Review and Meta-Analysis. J. Clin. Endocrinol. Metab..

[109] Song Y., Wang L., Pittas A.G., Del Gobbo L.C., Zhang C., Manson J.E., Hu F.B. (2013). Blood 25-hydroxy vitamin D levels and incident type 2 diabetes: A meta-analysis of prospective studies. Diabetes Care.

[110] Ekmekcioglu C., Haluza D., Kundi M. (2017). 25-Hydroxyvitamin D Status and Risk for Colorectal Cancer and Type 2 Diabetes Mellitus: A Systematic Review and Meta-Analysis of Epidemiological Studies. Int. J. Environ. Res. Public Health.

[111] Dawson-Hughes B., Staten M.A., Knowler W.C., Nelson J., Vickery E.M., LeBlanc E.S., Neff L.M., Park J., Pittas A.G. (2020). Intratrial Exposure to Vitamin D and New-Onset Diabetes Among Adults with Prediabetes: A Secondary Analysis from the Vitamin D and Type 2 Diabetes (D2d) Study. Diabetes Care.

[112] Lu L., Bennett D.A., Millwood I.Y., Parish S., McCarthy M.I., Mahajan A., Lin X., Bragg F., Guo Y., Holmes M.V. (2018). Association of vitamin D with risk of type 2 diabetes: A Mendelian randomisation study in European and Chinese adults. PLOS Med..

[113] Xu Y., Zhou Y., Liu J., Wang C., Qu Z., Wei Z., Zhou D. (2020). Genetically increased circulating 25(OH)D level reduces the risk of type 2 diabetes in subjects with deficiency of vitamin D: A large-scale Mendelian randomization study. Medicine.

[114] Hadi H.A.R., Carr C.S., Al Suwaidi J. (2005). Endothelial Dysfunction: Cardiovascular Risk Factors, Therapy, and Outcome. Vasc. Health Risk Manag..

[115] Latic N., Erben R.G. (2020). Vitamin D and Cardiovascular Disease, with Emphasis on Hypertension, Atherosclerosis, and Heart Failure. Int. J. Mol. Sci..

[116] Wong M.S.K., Delansorne R., Man R.Y.K., Svenningsen P., Vanhoutte P.M. (2010). Chronic treatment with vitamin D lowers arterial blood pressure and reduces endothelium-dependent contractions in the aorta of the spontaneously hypertensive rat. Am. J. Physiol. Circ. Physiol..

[117] Kassi E., Adamopoulos C., Basdra E.K., Papavassiliou A.G. (2013). Role of Vitamin D in Atherosclerosis. Circulation.

[118] Ford E.S., Ajani U.A., McGuire L.C., Liu S. (2005). Concentrations of Serum Vitamin D and the Metabolic Syndrome Among U.S. Adults. Diabetes Care.

[119] Forman J.P., Giovannucci E., Holmes M.D., Bischoff-Ferrari H.A., Tworoger S.S., Willett W.C., Curhan G.C. (2007). Plasma 25-Hydroxyvitamin D Levels and Risk of Incident Hypertension. Hypertension.

[120] Mokhtari E., Hajhashemy Z., Saneei P. (2022). Serum Vitamin D Levels in Relation to Hypertension and Pre-hypertension in Adults: A Systematic Review and Dose–Response Meta-Analysis of Epidemiologic Studies. Front. Nutr..

[121] Zhang D., Cheng C., Wang Y., Sun H., Yu S., Xue Y., Liu Y., Li W., Li X. (2020). Effect of Vitamin D on Blood Pressure and Hypertension in the General Population: An Update Meta-Analysis of Cohort Studies and Randomized Controlled Trials. Prev. Chronic Dis..

[122] Liu D., Fernandez B.O., Hamilton A., Lang N.N., Gallagher J.M., Newby D.E., Feelisch M., Weller R.B. (2014). UVA Irradiation of Human Skin Vasodilates Arterial Vasculature and Lowers Blood Pressure Independently of Nitric Oxide Synthase. J. Investig. Dermatol..

[123] Forman J.P., Scott J.B., Ng K., Drake B., Suarez E.G., Hayden D.L., Bennett G.G., Chandler P., Hollis B.W., Emmons K.M. (2013). Effect of Vitamin D Supplementation on Blood Pressure in Blacks. Hypertension.

[124] Pilz S., Gaksch M., Kienreich K., Grübler M., Verheyen N., Fahrleitner-Pammer A., Treiber G., Drechsler C., ó Hartaigh B., Obermayer-Pietsch B. (2015). Effects of vitamin D on blood pressure and cardiovascular risk factors: A randomized controlled trial. Hypertension.

[125] Theiler-Schwetz V., Trummer C., Grübler M.R., Keppel M.H., Zittermann A., Tomaschitz A., Karras S.N., März W., Pilz S., Gängler S. (2022). Effects of Vitamin D Supplementation on 24-Hour Blood Pressure in Patients with Low 25-Hydroxyvitamin D Levels: A Randomized Controlled Trial. Nutrients.

[126] Vimaleswaran K.S., Cavadino A., Berry D.J., Jorde R., Dieffenbach A.K., Lu C., Alves A.C., Heerspink H.J.L., Tikkanen E., Eriksson J. (2014). Association of vitamin D status with arterial blood pressure and hypertension risk: A mendelian randomisation study. Lancet Diabetes Endocrinol..

[127] Zittermann A., Iodice S., Pilz S., Grant W., Bagnardi V., Gandini S. (2012). Vitamin D deficiency and mortality risk in the general population: A meta-analysis of prospective cohort studies. Am. J. Clin. Nutr..

[128] Garland C.F., Kim J.J., Mohr S.B., Gorham E.D., Grant W.B., Giovannucci E.L., Baggerly L., Hofflich H., Ramsdell J.W., Zeng K. (2014). Meta-analysis of All-Cause Mortality According to Serum 25-Hydroxyvitamin D. Am. J. Public Health.

[129] Gaksch M., Jorde R., Grimnes G., Joakimsen R., Schirmer H., Wilsgaard T., Mathiesen E.B., Njølstad I., Løchen M.-L., März W. (2017). Vitamin D and mortality: Individual participant data meta-analysis of standardized 25-hydroxyvitamin D in 26916 individuals from a European consortium. PLoS ONE.

[130] Afzal S., Brøndum-Jacobsen P., E Bojesen S., Nordestgaard B.G. (2014). Genetically low vitamin D concentrations and increased mortality: Mendelian randomisation analysis in three large cohorts. BMJ.

[131] Sofianopoulou E., Kaptoge S.K., Afzal S., Jiang T., Gill D., Gundersen T.E., Bolton T.R., Allara E., Arnold M.G., Mason A.M. (2021). Estimating dose-response relationships for vitamin D with coronary heart disease, stroke, and all-cause mortality: Observational and Mendelian randomisation analyses. Lancet Diabetes Endocrinol..

[132] Cantorna M.T., Snyder L., Lin Y.-D., Yang L. (2015). Vitamin D and 1,25(OH)2D Regulation of T cells. Nutrients.

[133] Cannell J.J., Vieth R., Umhau J.C., Holick M.F., Grant W.B., Madronich S., Garland C.F., Giovannucci E. (2006). Epidemic influenza and vitamin D. Epidemiol. Infect..

[134] Shaman J., Kohn M. (2009). Absolute humidity modulates influenza survival, transmission, and seasonality. Proc. Natl. Acad. Sci. USA.

[135] Ianevski A., Zusinaite E., Shtaida N., Kallio-Kokko H., Valkonen M., Kantele A., Telling K., Lutsar I., Letjuka P., Metelitsa N. (2019). Low Temperature and Low UV Indexes Correlated with Peaks of Influenza Virus Activity in Northern Europe during 2010–2018. Viruses.

[136] Urashima M., Segawa T., Okazaki M., Kurihara M., Wada Y., Ida H. (2010). Randomized trial of vitamin D supplementation to prevent seasonal influenza A in schoolchildren. Am. J. Clin. Nutr..

[137] Panza F., La Montagna M., Lampignano L., Zupo R., Bortone I., Castellana F., Sardone R., Borraccino L., Dibello V., Resta E. (2021). Vitamin D in the development and progression of Alzheimer’s disease: Implications for clinical management. Expert Rev. Neurother..

[138] Jayedi A., Rashidy-Pour A., Shab-Bidar S. (2019). Vitamin D status and risk of dementia and Alzheimer’s disease: A meta-analysis of dose-response. Nutr. Neurosci..

[139] Chai B., Gao F., Wu R., Dong T., Gu C., Lin Q., Zhang Y. (2019). Vitamin D deficiency as a risk factor for dementia and Alzheimer’s disease: An updated meta-analysis. BMC Neurol..

[140] Olsson E., Byberg L., Karlström B., Cederholm T., Melhus H., Sjögren P., Kilander L. (2017). Vitamin D is not associated with incident dementia or cognitive impairment: An 18-y follow-up study in community-living old men. Am. J. Clin. Nutr..

[141] Littlejohns T.J., Kos K., Henley W.E., Kuzma E., Llewellyn D.J. (2016). Vitamin D and Dementia. J. Prev. Alzheimer’s Dis..

[142] Feart C., Helmer C., Merle B., Herrmann F.R., Annweiler C., Dartigues J., Delcourt C., Samieri C. (2017). Associations of lower vitamin D concentrations with cognitive decline and long-term risk of dementia and Alzheimer’s disease in older adults. Alzheimer’s Dement..

[143] Knekt P., Sääksjärvi K., Järvinen R., Marniemi J., Männistö S., Kanerva N., Heliövaara M. (2014). Serum 25-Hydroxyvitamin D Concentration and Risk of Dementia. Epidemiology.

[144] Licher S., de Bruijn R.F., Wolters F.J., Zillikens M.C., Ikram M.A. (2017). Vitamin D and the Risk of Dementia: The Rotterdam Study. J. Alzheimer’s Dis..

[145] Schneider A.L., Lutsey P.L., Alonso A., Gottesman R.F., Sharrett A.R., Carson K.A., Gross M., Post W.S., Knopman D.S., Mosley T.H. (2014). Vitamin D and cognitive function and dementia risk in a biracial cohort: The ARIC Brain MRI Study. Eur. J. Neurol..

[146] Karakis I., Pase M.P., Beiser A., Booth S.L., Jacques P.F., Rogers G., DeCarli C., Vasan R.S., Wang T.J., Himali J.J. (2016). Association of Serum Vitamin D with the Risk of Incident Dementia and Subclinical Indices of Brain Aging: The Framingham Heart Study. J. Alzheimer’s Dis..

[147] Mokry L.E., Ross S., Morris J.A., Manousaki D., Forgetta V., Richards J.B. (2016). Genetically decreased vitamin D and risk of Alzheimer disease. Neurology.

[148] Wang L., Qiao Y., Zhang H., Zhang Y., Hua J., Jin S., Liu G. (2020). Circulating Vitamin D Levels and Alzheimer’s Disease: A Mendelian Randomization Study in the IGAP and UK Biobank. J. Alzheimer’s Dis..

[149] Meng L., Wang Z., Ming Y.-C., Shen L., Ji H.-F. (2022). Are micronutrient levels and supplements causally associated with the risk of Alzheimer’s disease? A two-sample Mendelian randomization analysis. Food Funct..

[150] Zhang H., Wang T., Han Z., Wang L., Zhang Y., Wang L., Liu G. (2020). Impact of Vitamin D Binding Protein Levels on Alzheimer’s Disease: A Mendelian Randomization Study. J. Alzheimer’s Dis..

[151] De Haan P., Klein H.C., Hart B.A. (2017). Autoimmune Aspects of Neurodegenerative and Psychiatric Diseases: A Template for Innovative Therapy. Front. Psychiatry.

[152] Kouba B.R., Camargo A., Gil-Mohapel J., Rodrigues A.L.S. (2022). Molecular Basis Underlying the Therapeutic Potential of Vitamin D for the Treatment of Depression and Anxiety. Int. J. Mol. Sci..

[153] Luthold R.V., Fernandes G.R., Franco-De-Moraes A.C., Folchetti L.G., Ferreira S.R.G. (2017). Gut microbiota interactions with the immunomodulatory role of vitamin D in normal individuals. Metabolism.

[154] Xie F., Huang T., Lou D., Fu R., Ni C., Hong J., Ruan L. (2022). Effect of vitamin D supplementation on the incidence and prognosis of depression: An updated meta-analysis based on randomized controlled trials. Front. Public Health.

[155] Li H., Sun D., Wang A., Pan H., Feng W., Ng C.H., Ungvari G.S., Tao L., Li X., Wang W. (2019). Serum 25-Hydroxyvitamin D Levels and Depression in Older Adults: A Dose–Response Meta-Analysis of Prospective Cohort Studies. Am. J. Geriatr. Psychiatry.

[156] Mikola T., Marx W., Lane M.M., Hockey M., Loughman A., Rajapolvi S., Rocks T., O’Neil A., Mischoulon D., Valkonen-Korhonen M. (2022). The effect of vitamin D supplementation on depressive symptoms in adults: A systematic review and meta-analysis of randomized controlled trials. Crit. Rev. Food Sci. Nutr..

[157] Luo C.-W., Chen S.-P., Chiang C.-Y., Wu W.-J., Chen C.-J., Chen W.-Y., Kuan Y.-H. (2022). Association between Ultraviolet B Exposure Levels and Depression in Taiwanese Adults: A Nested Case–Control Study. Int. J. Environ. Res. Public Health.

[158] Mulugeta A., Lumsden A., Hyppönen E. (2020). Relationship between Serum 25(OH)D and Depression: Causal Evidence from a Bi-Directional Mendelian Randomization Study. Nutrients.

[159] Wagner C.L., Hollis B.W. (2018). The Implications of Vitamin D Status During Pregnancy on Mother and her Developing Child. Front. Endocrinol..

[160] Aguilar-Cordero M., Lasserrot-Cuadrado A., Mur-Villar N., León-Ríos X., Rivero-Blanco T., Pérez-Castillo I. (2020). Vitamin D, preeclampsia and prematurity: A systematic review and meta-analysis of observational and interventional studies. Midwifery.

[161] Gernand A.D., Bodnar L.M., A Klebanoff M., Parks W.T., Simhan H.N. (2013). Maternal serum 25-hydroxyvitamin D and placental vascular pathology in a multicenter US cohort. Am. J. Clin. Nutr..

[162] Merewood A., Mehta S.D., Chen T.C., Bauchner H., Holick M.F. (2009). Association between Vitamin D Deficiency and Primary Cesarean Section. J. Clin. Endocrinol. Metab..

[163] Scholl T.O., Chen X., Stein P. (2012). Maternal Vitamin D Status and Delivery by Cesarean. Nutrients.

[164] Hu L., Zhang Y., Wang X., You L., Xu P., Cui X., Zhu L., Ji C., Guo X., Wen J. (2018). Maternal Vitamin D Status and Risk of Gestational Diabetes: A Meta-Analysis. Cell. Physiol. Biochem..

[165] Milajerdi A., Abbasi F., Mousavi S.M., Esmaillzadeh A. (2021). Maternal vitamin D status and risk of gestational diabetes mellitus: A systematic review and meta-analysis of prospective cohort studies. Clin. Nutr..

[166] Wagner C., Baggerly C., McDonnell S., Hamilton S., Winkler J., Warner G., Rodriguez C., Shary J., Smith P., Hollis B. (2015). Post-hoc comparison of vitamin D status at three timepoints during pregnancy demonstrates lower risk of preterm birth with higher vitamin D closer to delivery. J. Steroid Biochem. Mol. Biol..

[167] Zhou S.-S., Tao Y.-H., Huang K., Zhu B.-B., Tao F.-B. (2017). Vitamin D and risk of preterm birth: Up-to-date meta-analysis of randomized controlled trials and observational studies. J. Obstet. Gynaecol. Res..

[168] Whitehouse A.J.O., Holt B.J., Serralha M., Holt P.G., Kusel M.M.H., Hart P.H. (2012). Maternal Serum Vitamin D Levels During Pregnancy and Offspring Neurocognitive Development. Pediatrics.

[169] Garcia-Serna A.M., Morales E. (2020). Neurodevelopmental effects of prenatal vitamin D in humans: Systematic review and meta-analysis. Mol. Psychiatry.

[170] Shi D., Wang D., Meng Y., Chen J., Mu G., Chen W. (2021). Maternal vitamin D intake during pregnancy and risk of asthma and wheeze in children: A systematic review and meta-analysis of observational studies. J. Matern. Neonatal Med..

[171] Robinson C.J., Alanis M.C., Wagner C.L., Hollis B.W., Johnson D.D. (2010). Plasma 25-hydroxyvitamin D levels in early-onset severe preeclampsia. Am. J. Obstet. Gynecol..

[172] Serrano-Diaz N.C., Gamboa-Delgado E.M., Dominguez-Urrego C.L., Vesga-Varela A.L., Serrano-Gomez S.E., Quintero-Lesmes D.C. (2018). Vitamin D and risk of preeclampsia: A systematic review and meta-analysis. Biomedica.

[173] Gallo S., McDermid J.M., Al-Nimr R.I., Hakeem R., Moreschi J.M., Pari-Keener M., Stahnke B., Papoutsakis C., Handu D., Cheng F.W. (2020). Vitamin D Supplementation during Pregnancy: An Evidence Analysis Center Systematic Review and Meta-Analysis. J. Acad. Nutr. Diet..

[174] Palacios C., Kostiuk L.K., Pena-Rosas J.P. (2019). Vitamin D supplementation for women during pregnancy. Cochrane Database Syst. Rev..

[175] Saha S., Saha S. (2020). A comparison of the risk of cesarean section in gestational diabetes mellitus patients supplemented antenatally with vitamin D containing supplements versus placebo: A systematic review and meta-analysis of double-blinded randomized controlled trials. J. Turk. Ger. Gynecol. Assoc..

[176] Fogacci S., Fogacci F., Banach M., Michos E.D., Hernandez A.V., Lip G.Y., Blaha M.J., Toth P.P., Borghi C., Cicero A.F.G. (2020). Vitamin D supplementation and incident preeclampsia: A systematic review and meta-analysis of randomized clinical trials. Clin. Nutr..

[177] Kinshella M.-L., Omar S., Scherbinsky K., Vidler M., Magee L., von Dadelszen P., Moore S., Elango R. (2021). The PRECISE Conceptual Framework Working Group Effects of Maternal Nutritional Supplements and Dietary Interventions on Placental Complications: An Umbrella Review, Meta-Analysis and Evidence Map. Nutrients.

[178] Godfrey K.M., Costello P.M., Lillycrop K.A. (2016). Development, Epigenetics and Metabolic Programming. Prev. Asp. Early Nutr..

[179] Moon R.J., Curtis E.M., Woolford S.J., Ashai S., Cooper C., Harvey N.C. (2021). The importance of maternal pregnancy vitamin D for offspring bone health: Learnings from the MAVIDOS trial. Ther. Adv. Musculoskelet. Dis..

[180] Ross A.C., Manson J.E., Abrams S.A., Aloia J.F., Brannon P.M., Clinton S.K., Durazo-Arvizu R.A., Gallagher J.C., Gallo R.L., Jones G. (2011). The 2011 Report on Dietary Reference Intakes for Calcium and Vitamin D from the Institute of Medicine: What Clinicians Need to Know. J. Clin. Endocrinol. Metab..

[181] Toner C.D., Davis C.D., Milner J.A. (2010). The Vitamin D and Cancer Conundrum: Aiming at a Moving Target. J. Am. Diet. Assoc..

[182] Sempos C.T., Durazo-Arvizu R.A., Dawson-Hughes B., Yetley E.A., Looker A.C., Schleicher R.L., Cao G., Burt V., Kramer H., Bailey R.L. (2013). Is There a Reverse J-Shaped Association Between 25-Hydroxyvitamin D and All-Cause Mortality? Results from the U.S. Nationally Representative NHANES. J. Clin. Endocrinol. Metab..

[183] Wagner C.L., Hulsey T.C., Fanning D., Ebeling M., Hollis B.W. (2006). High-Dose Vitamin D_3_ Supplementation in a Cohort of Breastfeeding Mothers and Their Infants: A 6-Month Follow-Up Pilot Study. Breastfeed. Med..

[184] Hill A.B. (1965). The Environment and Disease: Association or Causation?. Proc. R. Soc. Med..

[185] Potischman N., Weed D.L. (1999). Causal criteria in nutritional epidemiology. Am. J. Clin. Nutr..

[186] Grant W.B. (2009). How strong is the evidence that solar ultraviolet B and vitamin D reduce the risk of cancer? An examination using Hill’s criteria for causality. Derm.-Endocrinol..

[187] Mohr S.B., Gorham E.D., Alcaraz J.E., Kane C.I., Macera C.A., Parsons J.K., Wingard D.L., Garland C.F. (2012). Does the evidence for an inverse relationship between serum vitamin D status and breast cancer risk satisfy the Hill criteria?. Derm.-Endocrinol..

[188] Robsahm T.E., Schwartz G.G., Tretli S. (2013). The Inverse Relationship between 25-Hydroxyvitamin D and Cancer Survival: Discussion of Causation. Cancers.

[189] Grant W.B. (2020). Review of Recent Advances in Understanding the Role of Vitamin D in Reducing Cancer Risk: Breast, Colorectal, Prostate, and Overall Cancer. Anticancer Res..

[190] Weyland P.G., Grant W.B., Howie-Esquivel J. (2014). Does Sufficient Evidence Exist to Support a Causal Association between Vitamin D Status and Cardiovascular Disease Risk? An Assessment Using Hill’s Criteria for Causality. Nutrients.

[191] Wimalawansa S.J. (2018). Vitamin D and cardiovascular diseases: Causality. J. Steroid Biochem. Mol. Biol..

[192] Walsh J.B., McCartney D.M., Laird É., McCarroll K., Byrne D.G., Healy M., O’Shea P.M., Kenny R.A., Faul J.L. (2022). Understanding a Low Vitamin D State in the Context of COVID-19. Front. Pharmacol..

[193] Annweiler C. (2016). Vitamin D in dementia prevention. Ann. N. Y. Acad. Sci..

[194] Altieri B., Grant W.B., Della Casa S., Orio F., Pontecorvi A., Colao A., Sarno G., Muscogiuri G. (2017). Vitamin D and pancreas: The role of sunshine vitamin in the pathogenesis of diabetes mellitus and pancreatic cancer. Crit. Rev. Food Sci. Nutr..

[195] Zipitis C.S., Akobeng A.K. (2008). Vitamin D supplementation in early childhood and risk of type 1 diabetes: A systematic review and meta-analysis. Arch. Dis. Child..

[196] Giovannoni G., Ebers G. (2007). Multiple sclerosis: The environment and causation. Curr. Opin. Neurol..

[197] Hanwell H.E., Banwell B. (2011). Assessment of evidence for a protective role of vitamin D in multiple sclerosis. Biochim. Biophys. Acta (BBA) Mol. Basis Dis..

[198] Uwitonze A.M., Murererehe J., Ineza M.C., Harelimana E.I., Nsabimana U., Uwambaye P., Gatarayiha A., Haq A., Razzaque M.S. (2018). Effects of vitamin D status on oral health. J. Steroid Biochem. Mol. Biol..

[199] Grant W.B., Boucher B.J. (2010). Are Hill’s criteria for causality satisfied for vitamin D and periodontal disease?. Derm.-Endocrinol..

[200] Carlberg C. (2019). Nutrigenomics of Vitamin D. Nutrients.

